# Mating and blood-feeding induce transcriptome changes in the spermathecae of the yellow fever mosquito *Aedes aegypti*

**DOI:** 10.1038/s41598-020-71904-z

**Published:** 2020-09-10

**Authors:** Carolina Camargo, Yasir H. Ahmed-Braimah, I. Alexandra Amaro, Laura C. Harrington, Mariana F. Wolfner, Frank W. Avila

**Affiliations:** 1 Max Planck Tandem Group in Mosquito Reproductive Biology, Universidad de Antioquia, Complejo RutaN, Calle 67 #52-20, Laboratory 4-166, 050010 Medellín, Colombia; 2 Department of Biology, Syracuse University, Syracuse, NY 13244 USA; 3 Department of Entomology, Cornell University, Ithaca, NY 14850 USA; 4 Department of Molecular Biology and Genetics, Cornell University, Ithaca, NY 14850 USA

**Keywords:** Molecular biology, Transcriptomics

## Abstract

*Aedes aegypti* mosquitoes are the primary vectors of numerous viruses that impact human health. As manipulation of reproduction has been proposed to suppress mosquito populations, elucidation of biological processes that enable males and females to successfully reproduce is necessary. One essential process is female sperm storage in specialized structures called spermathecae. *Aedes aegypti* females typically mate once, requiring them to maintain sperm viably to fertilize eggs they lay over their lifetime. Spermathecal gene products are required for *Drosophila* sperm storage and sperm viability, and a spermathecal-derived heme peroxidase is required for long-term *Anopheles gambiae* fertility. Products of the *Ae. aegypti* spermathecae, and their response to mating, are largely unknown. Further, although female blood-feeding is essential for anautogenous mosquito reproduction, the transcriptional response to blood-ingestion remains undefined in any reproductive tissue. We conducted an RNAseq analysis of spermathecae from unfed virgins, mated only, and mated and blood-fed females at 6, 24, and 72 h post-mating and identified significant differentially expressed genes in each group at each timepoint. A blood-meal following mating induced a greater transcriptional response in the spermathecae than mating alone. This study provides the first view of elicited mRNA changes in the spermathecae by a blood-meal in mated females.

## Introduction

*Aedes aegypti* mosquitoes are responsible for the transmission of the dengue^1^, chikungunya^2^, Zika^3^ and yellow fever viruses^4,5^. Due to the public health burden of these diseases, it is crucial to control their spread in order to minimize their impact^6^. One approach is to control mosquito populations^7^. To date, *Ae. aegypti* control has primarily focused on insecticides to reduce larval and adult populations^8^. However, insecticide use is problematic due to the rapid evolution of insecticide resistance^8,9^ and the potential harm to ecosystems^10,11^. Disruption or manipulation of mosquito reproduction is an alternative to chemical control to suppress populations and/or reduce vectorial capacity^6^. Introduction of transgenic^12^ or *Wolbachia* infected^13,14^ *Ae. aegypti* is currently being tested to suppress or replace native populations. To aid such strategies, and to develop complementary methods, it is imperative to identify novel targets to reduce the fertility of this vector species, and to elucidate the biological processes that enable *Ae. aegypti* males and females to successfully reproduce.

Mosquito reproduction depends on the coordination of physiological and behavioral processes that occur in mated females. For *Ae. aegypti*, these processes may include increased host-seeking and blood-feeding behaviors, egg development rates, and sperm storage^15,16^. As *Ae. aegypti* females usually mate only once^17–19^, the storage and maintenance of sperm are fundamental processes that maximize reproductive output and fitness^20^. During mating in *Ae. aegypti*, sperm are deposited into the bursa of the female reproductive tract. They quickly concentrate at the spermathecal vestibule and migrate to the sperm storage organs (referred to as spermathecae), where they are stored long-term^21^. The environment of the spermathecae needs to be conducive to maintain sperm viably until they are released to fertilize eggs^21–23^. Sperm storage gives mosquitoes the capacity for high reproductive output as mating and oviposition are asynchronous, allowing females to be reproductively successful even with uncertain mating opportunities^24,25^.

The regulation of sperm entry into, protection within, and release from the storage organs requires male and female-derived molecules^26–28^. While emphasis has been placed on male-derived seminal proteins and their sperm storage effects, female-specific molecules that are required for proper sperm use are largely unknown^15,16,29^. Studies in different insect taxa have shown that spermathecae have associated secretory cells (spermathecal secretory cells or SSCs) that produce proteins and other molecules that function in sperm storage^25,30,31^, with several SSC genes regulated by mating^27,32,33^. In *Drosophila*, genetic ablation of the SSCs impairs sperm motility, ovulation, egg-laying, and sperm entry into storage^27,34,35^. In social insects such as the honeybee *Apis melifera*^36^ and *Crematogaster osakensis* ant queens^37^, spermathecal secretions enhance sperm viability and are involved in the long-term maintenance of stored sperm. Moreover, spermathecal secretions of *Atta colombica* leafcutter ant queens halt competition between rival male ejaculates and enhance sperm viability^38^. In *Anopheles gambiae*, the spermathecal-derived heme peroxidase HPX15 is required for long-term female fertility^23^. Transcriptional and proteomic profiles of the sperm storage organs after mating in *Drosophila*^32,33^, *Apis*^39^, *Crematogaster*^37^ and *Anopheles*^23^ have identified molecules involved in the immune response, carbohydrate and lipid metabolism, cellular transport and oxidative stress that might play protective roles for sperm and/or mediate female post-mating processes.

*Aedes aegypti* spermathecae have associated secretory cells^21,25,40^. While gene expression in this tissue has been described at 7 days post-eclosion^31^, the transcriptional response of the spermathecae in the first hours and days after mating is unknown. Alfonso-Parra et al.^41^ identified large-scale transcriptional changes in lower reproductive tract tissues (i.e. reproductive tract minus ovaries) of *Ae. aegypti* females at 6 and 24 h post-mating. In *D. melanogaster*, several spermathecal genes are significantly differentially expressed at 3 and 6 h post-mating^32^. Furthermore, although blood-feeding is required for oocyte development^22^, studies examining the effect of blood-ingestion on transcriptional change in *Ae. aegypti* are limited to evaluation of whole bodies^42^, abdomens^43^, midgut tissues^44^, muscle mitochondria^45^, maxillary palps^46^, or on specific functions such as amino acid transport in the gut^47^. Despite the strong link between female blood-ingestion and reproduction in mosquitoes, no studies have described the transcriptional response of the spermathecae—or any reproductive tract tissue—to blood-feeding.

In this study, we used short-read mRNA sequencing (RNAseq) to analyze the spermathecae of three types of female: virgins, mated only and mated and subsequently blood-fed; the latter are referred to hereafter as Not Blood-Fed (NBF) and Blood-Fed (BF), respectively. Spermathecae from each group were assessed at 6, 24, and 72 h post-mating to measure transcript abundance levels shortly after mating and until females typically commence egg-laying. Unmated females were used as the baseline controls in our analysis. At all timepoints, we identified genes that show significant differences in transcript abundance compared to virgin controls. Mating followed by blood-ingestion had a larger effect than mating alone on spermathecal transcript abundance. Furthermore, we found that genes with transmembrane transport and ion transport activity represent the main enriched functional classes after mating. Blood-feeding in females altered several transcripts that encode proteins active in metabolic processes and amino-acid biosynthesis, which could be related to energy provisioning processes in *Ae. aegypti* spermathecae. This study identifies genes that are expressed in the sperm storage organs that are likely required for *Ae. aegypti* reproduction and have the potential for use to inhibit female fertility in the field.

## Results and discussion

Mating and blood-feeding in *Ae. aegypti* result in physiological and behavioral changes that primarily act to facilitate progeny production^15,16,42^. While seminal proteins induce female post-mating changes in insects^15^, including *Ae. aegypti*^17,48,49^, the molecular role of spermathecal gene products in fertility are not well-understood. Spermathecal proteins are essential for optimal fertility in *D. melanogaster*^27,33–35^ and *An. gambiae*^23^. In mosquitoes and most insects, spermathecae function as long-term storage compartments for sperm after insemination^21,23,25,31^. Here, we report transcript abundance profiles of the spermathecae at three distinct timepoints after mating and blood-feeding to understand the molecular mechanisms that allow females to maintain viable sperm.

**Figure 1:**
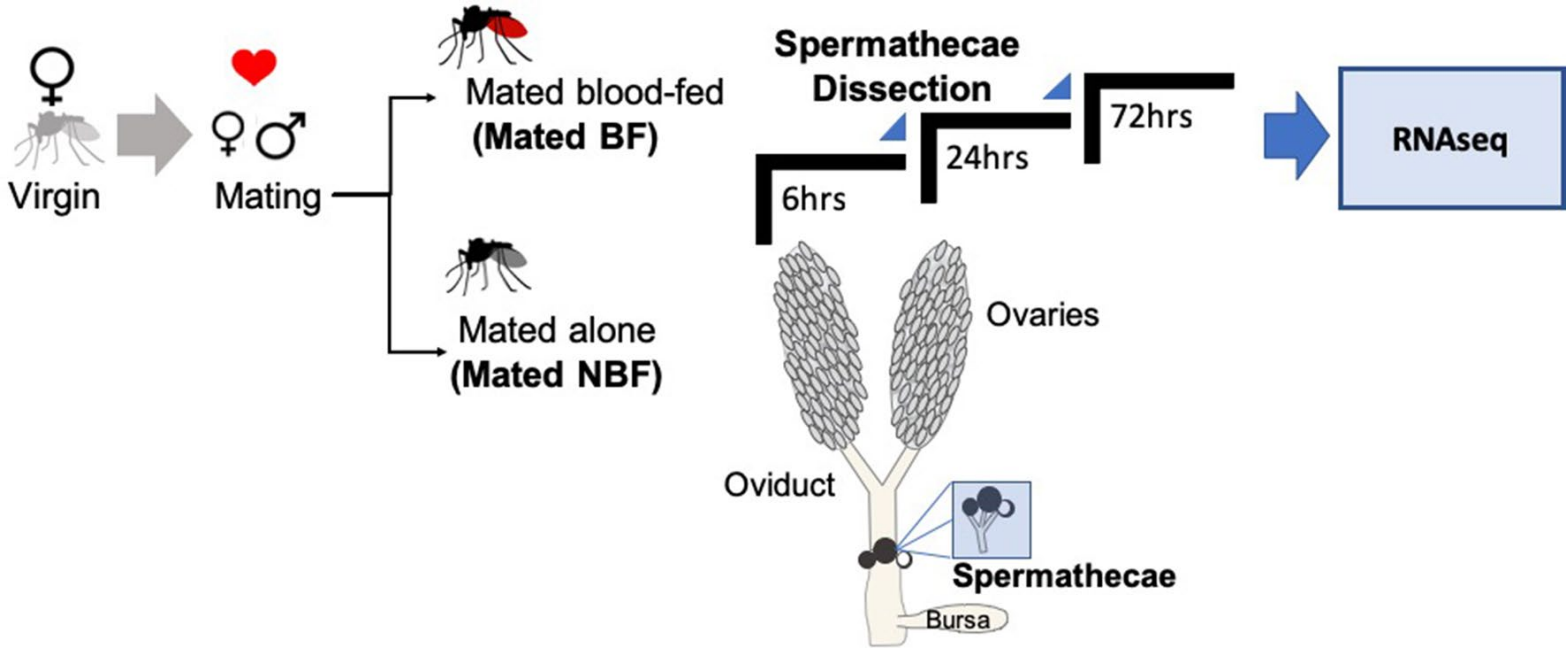
Schematic of spermathecae sample collection from virgin, NBF, and BF females. Spermathecae samples from mated females were dissected at the specified post-mating timepoints.

We generated mRNA libraries from spermathecae of virgin females, and NBF and BF females at 6, 24 and 72 h post-mating (Fig. 1). We designed an analysis pipeline to (1) compare transcript abundance in the spermathecae from NBF and BF females to virgin females irrespective of post-mating timepoint, (2) compare transcript abundance in the spermathecae from NBF and BF females to virgin females at each timepoint (6, 24 and 72 h), and (3) to directly compare post-mating NBF and BF females at each timepoint. Transcripts that exhibited a ≥ twofold change in abundance with a false discovery rate (FDR) < 0.05 were considered significantly differentially abundant. Normalization of transcripts was performed before differential expression analysis (Figure S1).

### Mating alters the transcript profile in the spermathecae

**Figure 2:**
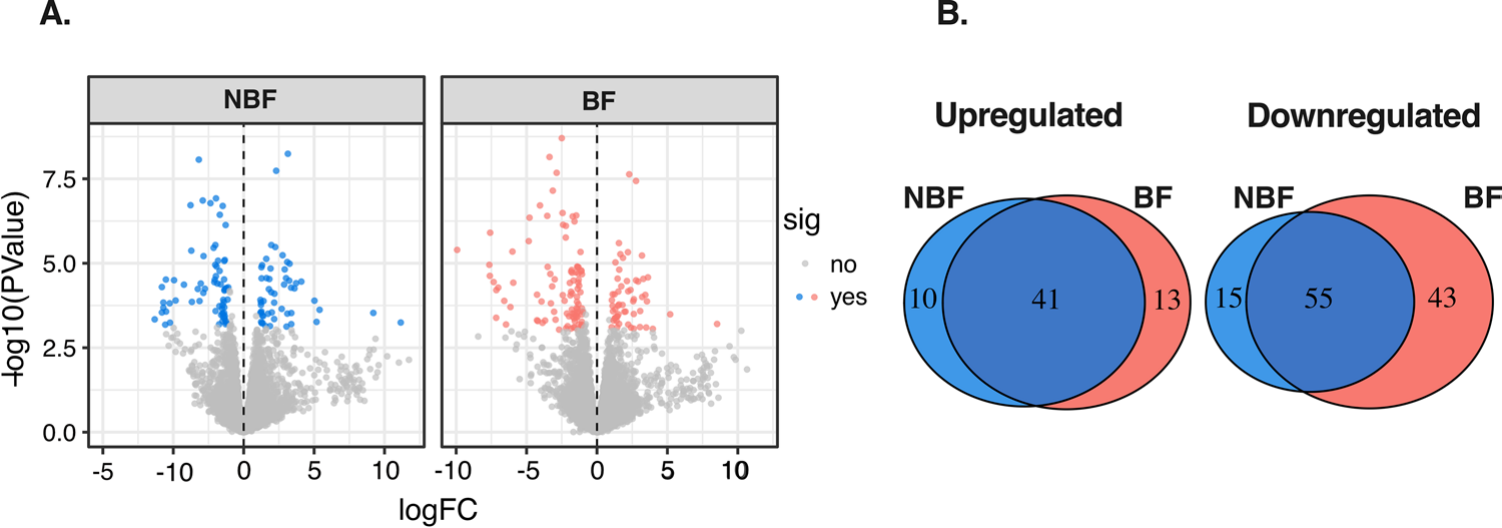
Differential gene expression profiles of NBF and BF females at all timepoints. (**A**) Volcano plot showing gene expression profiles of NBF females (left panel) and BF females (right panel). Values below zero represent down-regulated genes and values above zero up-regulated genes. Blue and red dots represent differentially expressed genes at *P* < 0.05 in NBF and BF samples, respectively. Grey dots represent genes that are not differentially expressed. (**B**) Venn diagrams representing the number of up- and down-regulated genes in NBF and BF females.

To investigate the global transcriptomic response to mating and blood-intake in spermathecal tissue, we first analyzed the combined post-mating timepoints. This approach reduced complexity, simplified the dimensionality of the data, and reduced the false discovery rate, enabling us to identify candidate transcripts in NBF and BF females. We found 275 transcripts that were significantly differentially expressed in spermathecae from both NBF and BF females compared to virgins (Fig. 2; Figure S2A–B; File S3)—107 up- and 168 down-regulated (Fig. 2A). We observed a higher number of differentially expressed transcripts in spermathecae from BF females (152 transcripts: 54 up- and 98 down-regulated; Fig. 2A, right panel), compared to NBF females (121 transcripts: 51 up- and 70 down-regulated; Fig. 2A, left panel). A similar number of transcripts were uniquely up-regulated in spermathecae from NBF and BF females; 10 and 13 transcripts, respectively (Fig. 2B). However, we observed a significant difference in the number of down-regulated genes in spermathecae from BF females compared to NBF females (DF = 1; χ^2^ = 33.5; *P* < 0.0001; Fig. 2B). These results indicate that mating and blood-feeding each induce a transcriptional response in the spermathecae. In hematophagous insects such as *Ae. aegypti*, blood intake causes significant physiological and metabolic constraints^50,51^. Thus, while spermathecae from BF females and NBF females overlap in their physiological and metabolic processes for sperm maintenance during storage, stress caused by a blood-meal might independently effect a transcriptional response in the spermathecae.

Regardless of female feeding status, sperm require energetic nourishment, protection from oxidative stress and a suitable pH environment within storage^20^. Our results show a total of 40 transcripts that are up-regulated in response to mating in the spermathecae of NBF and BF females (Fig. 2B; File S3). The most significant up-regulated genes in spermathecae from NBF and BF females correspond to proteins with roles in ion transport, such as bumetanide-sensitive sodium coupled cation-chloride cotransporter (AAEL009888) and sodium-dependent phosphate transporter protein (AAEL001656) (Fig. 2A; File S3). Ion transport genes are also regulated by mating in the spermathecae of social insects^29^ and in the *D. melanogaster* seminal receptacle^32^, a second sperm storage organ in this species, indicating the importance of ion balance in insect sperm storage. As insect cells have mechanisms to control pH through ion exchange (e.g. Na, H, K, etc.)^52,53^, the up-regulation of these genes might be related to the ionic balance and/or pH of the storage organs. Moreover, we found other transcripts potentially related to the regulation of the spermathecae’s osmotic environment, such as putative calcium ion binding transcripts troponin C (AAEL012114), EF-hand domain-containing protein (AAEL006006), and several genes whose products relate to transmembrane transport (AAEL027912, AAEL010883, AAEL024256, AAEL000488, AAEL012041, AAEL007979, AAEL012041, AAEL014311, AAEL020162) (File S3).

Sperm maintenance within storage requires energy provisioning substrates such as polysaccharides and lipoproteins^20^. Although energy substrates for sperm maintenance have not been identified in mosquitoes^54^, it is reasonable to hypothesize that energy for sperm nourishment may be provided by the spermathecal cells^21^. We found two transcripts in the spermathecae from both NBF and BF females that encode proteins that potentially function in sperm nourishment and energy metabolism: the oxysterol-binding protein related to lipid transport (AAEL018354) and phospholipase A1 (AAEL006982). In spermathecae from BF females, we found genes related to energy provisioning such as trehalase (AAEL009658) and transcripts related to amino-acid metabolism such as asparagine synthetase (AAEL015631) and cysteine dioxygenase (AAEL026357).

Moreover, during sperm storage, the imbalance of reactive oxygen species and antioxidants can cause oxidative stress^55^. Antioxidant genes and proteins have been reported in *A. mellifera*^39^ and *An. gambiae*^23^ spermathecae, and the *D. melanogaster*^32^ seminal receptacle. We found three transcripts with potential roles in oxidative stress in the BF and NBF female groups: egg-antigen (AAEL001094), the synaptic vesicle membrane protein VAT-1 homolog-like (AAEL019604), and a predicted oxidative stress-induced growth inhibitor (AAEL002835) (Fig. 2A; File S3). The egg-antigen belongs to the heat-shock protein family. Heat shock proteins have roles in reducing oxidative stress in other insects, including *Drososphila*^56^ and the honey bee^57^.

Differential expression analysis also identified several down-regulated transcripts in NBF and BF females. A total of 55 genes are significantly down-regulated in spermathecae from both NBF and BF females compared to virgins (Fig. 2B). The most significant down-regulated transcripts correspond to cytochrome P450 49a1 (AAEL008638), a zinc finger protein (AAEL004057), sodium/hydrogen exchanger 9B2 (AAEL011109) and nardilysin (AAEL010073) (File S3). Post-mating down-regulation of P450 genes—which encode proteins with several functions including oxidation catalysis of exogenous substances—occurs in the *D. melanogaster* sperm storage organs^32^. Regulation of P450 transcripts might minimize oxidative stress and suppress an immune response to protect sperm cells within storage^32,41,58^.

### Temporal transcriptional profile dynamics of the spermathecae in response to mating and blood-feeding

Physiological and structural changes in female reproductive tract tissue and/or stored sperm occurs in insects during and soon after mating ends^59–65^. In *D. melanogaster*, oviductal tissue remodeling^61^, uterine conformation^65,66^, and increased innervation of oviduct musculature^63^ are detectable in mated females during, or by 6 h after mating. In *An. gambiae*, permanent structural changes of the female reproductive tract are detectable at 8 h post-mating^62,67^. *Aedes aegypti* sperm undergo a coat modification within the spermathecae detectable at 4 h and complete by 24 h post-mating^64^. Mating responsive genes in the female reproductive tract potentially mediate post-mating tissue or sperm modification and/or coordinate activities with seminal proteins that are required for some of these changes^59,60,63^. Transcriptome studies of *Drosophila* female reproductive tract tissues show that peak differential gene expression occurs at 6 h post-mating^68,69^. In *An. gambiae*, the number of genes that experience a twofold or greater change in expression increases at each consecutive post-mating time point assessed (2, 6, and 24 h)^62^ and peak post-mating gene expression in the spermatheca occurs at 24 h^23^. In the *Ae. aegypti* lower reproductive tract, more genes are differentially expressed at 24 h than at 6 h post-mating^41^. To determine how *Ae. aegypti* spermathecae respond to mating and blood-feeding, we examined transcript abundance in the spermathecae at 6 and 24 h to uncover early changes. We also examined transcript abundance at 72 h, when oocytes for our mosquitoes had matured following a blood-meal and egg-laying/sperm release from storage commences.

**Figure 3:**
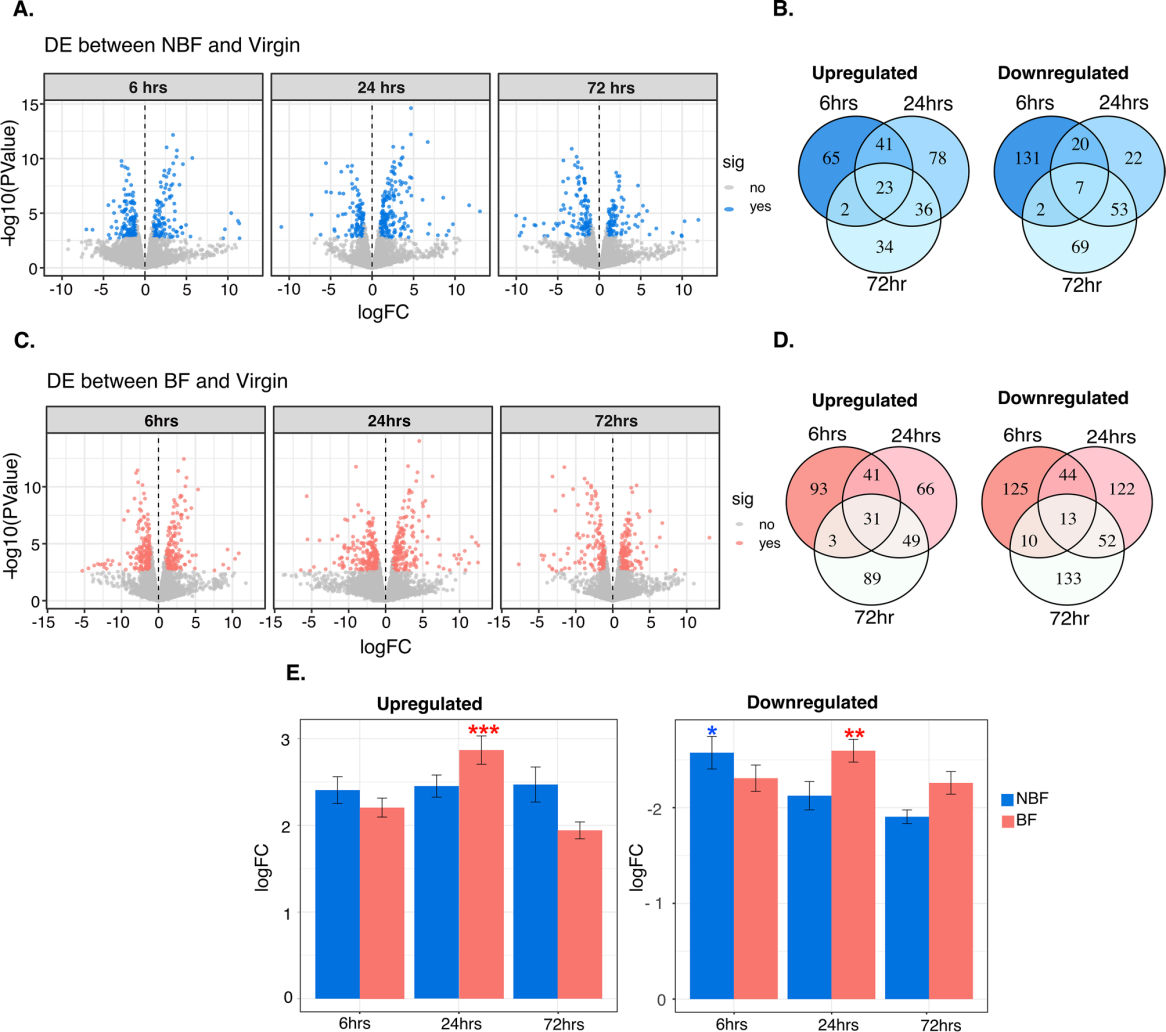
Differentially expressed transcripts at 6, 24 and 72 h post-mating in spermathecae from NBF and BF females. (**A**) Volcano plot showing total number of transcripts at each time point in NBF females. Blue dots represent significantly differentially expressed transcripts (*P* < 0.05; FDR < 0.05). (**B**) Venn diagram showing the intersection of transcripts in NBF females. (**C**) Volcano plot showing the total transcripts at each timepoint in BF females. Red dots represent significant differentially expressed transcripts. (**D**) Venn diagram showing the intersection of genes in BF females. (**E**) Relative abundance of transcripts in NBF and BF females at the indicated timepoint. Bars represent logFC average standard error for total differentially expressed transcripts. Significant differences between timepoints within female group are shown: ***P = 0.0001, **P = 0.001, *P = 0.01.

We observed a large number of differences in transcript abundance patterns across the three time points, with a total of 797 differentially expressed transcripts in spermathecae from NBF females compared to 1,158 transcripts in the BF group (Fig. 3; Figure S2D–I; File S4). In spermathecae from NBF females, the number of differentially expressed transcripts gradually decreased—291, 280 and 226—at 6, 24 and 72 h, respectively (Fig. 3A,B; File S4). In BF females, however, differential expression peaked at 24 h (418 transcripts), compared to 360 at 6 h, and 380 at 72 h (Fig. 3C,D; File S4).

The magnitude of the transcriptional response—the average log-fold change of the total number of differentially expressed transcripts compared to virgins—significantly differed between the NBF and BF groups in both up- and down-regulated transcripts across all timepoints (LM *F* = 8.89, *P* = 0.00015; Fig. 3E). In spermathecae from BF females, the magnitude of the transcriptional response was significantly higher at 24 h compared to 6 and 72 h for both up-regulated (*P* < 0.0001) and down-regulated transcripts (*P* = 0.001). However, the magnitude of the transcriptional response in spermathecae from NBF females was similar across the three evaluated timepoints for up-regulated transcripts (*P* > 0.05). Furthermore, a significant decrease in down-regulated transcripts occurred after the 6 h timepoint in NBF females (*P* = 0.01; Fig. 3E).

### Blood-feeding after mating induces a transcriptional response in the spermathecae

Blood-feeding by females is a requirement for anautogenous mosquito reproduction. However, the potential link between blood-feeding and the post-mating regulation of gene expression in reproductive tissues has not been explored. While mating induces a transcriptional response in reproductive tract tissues of female mosquitoes^23,31,41^, the extent to which this transcriptional response is mating- vs. blood-meal-induced is not yet known. The mating encounter site for *Ae. aegypti* is around the host—males intercept females as they come to blood feed^70^. Therefore, to examine post-mating changes in gene expression in a biologically-relevant situation, we identified spermathecal transcripts in females that had first mated and blood-fed shortly afterward (see Methods). As mating alone induces a transcriptional response in the spermathecae (Figs. 2A, 3A), our data do not permit determination of spermathecal transcripts regulated only by blood ingestion. Recurring blood-feeding events by mated females may regulate genes important for sperm viability or for egg-development, ovulation and/or egg-laying.

**Figure 4:**
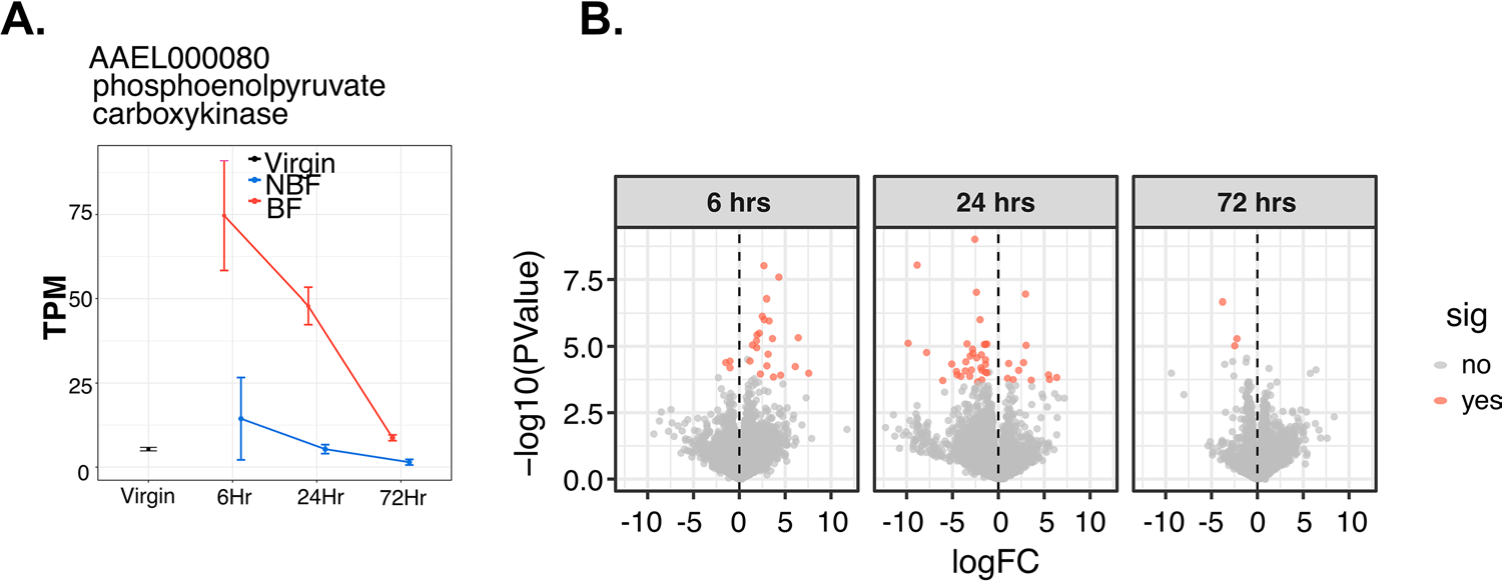
Differential gene expression profiles between spermathecae from NBF and BF females. (**A**) Expression of AAEL000080, the only significantly differentially expressed gene upon analysis of all timepoints combined (TPM = transcripts per million). (**B**) Volcano plot of transcripts identified at each post-mating timepoint. Orange dots represent significantly differentially expressed genes (*P* < 0.05). Values below zero (left side of the panel) represent down-regulated genes and points above zero (right side) represent up-regulated genes.

We compared transcript abundance between NBF and BF female spermatheca using: (1) the combined set of all timepoints for each female group, and (2) samples from each female type at each timepoint. We found only one differentially expressed gene when comparing the combined post-mating timepoints of BF spermatheca samples to NBF samples (FDR < 0.05; LogFC > 1; Figure S2C). This gene, phosphoenolpyruvate carboxy-kinase (PCK; AAEL000080), is associated with the generation of glucose from non-carbohydrate precursors, such as pyruvate and amino acids. The abundance pattern of this gene is significantly greater at 6 h in spermathecae from BF females and decreases at 24 and 72 h (Fig. 4A). Enzymes involved in glucose production, such as glucose-dehydrogenase in *D. melanogaster,* play a role in sperm nourishment and release from storage sites^71^. Thus, transcripts involved in catalytic reactions to produce energy in mosquito spermathecae might form a basis to discern the process of energy supply in the sperm storage organs.

Next, we examined transcripts at each post-mating timepoint. We observed a decreasing number of transcripts that were up-regulated by blood-feeding from 6 h (21 transcripts) to 24 h (10 transcripts) (Fig. 4B, Figure S2J–L; File S5). By 72 h we did not identify differentially abundant transcripts when comparing mated BF and mated NBF females (Fig. 4B). At 6 h, transcripts with the highest change in expression were related to energy metabolism: enoyl-CoA hydratase (AAEL003993), D-amino-acid oxidase (AAEL000213), and phosphoserine aminotransferase (AAEL012578) (File S5). At 24 h, vitellogenin-A1 (AAEL010434), vitellogenic carboxypeptidase (AAEL006563), and the general odorant-binding protein 99a (AAEL005772) were the most significantly up-regulated transcripts (logFC > 3, FDR < 0.01). Although vitellogenesis is activated by blood-feeding, vitellogenin is up-regulated > 200 fold in the *An. gambiae* spermatheca post-mating^62^. Yolk proteins are also detected in the *D. melanogaster* sperm storage organs^32^. Many up-regulated transcripts in BF females are related to energy metabolism and amino acid biosynthesis, suggesting that blood-feeding might elicit production of molecules for sperm nourishment and energy provision.

Only 3 transcripts were significantly down-regulated at 6 and 72 h. However, down-regulated transcripts drastically increased at 24 h, with a total of 32 differentially expressed transcripts (File S5). The majority of down-regulated transcripts at this time were peptidases (12 transcripts). The remaining 20 transcripts were uncharacterized proteins (9 transcripts), monoxygenases (2 transcripts), nucleic acid binding (2 transcripts), ion binding (2 transcripts), actin filament binding (2 transcripts), glucuronosyltransferase activity (1 transcript), putative cell wall protein (1 transcript) and integral component of membrane (1 transcript) (File S5). Peptidases are the main down-regulated transcripts in the *Ae. aegypti* lower reproductive tracts after mating^41^. Modulating peptidase expression post-mating might be important to avoid sperm degradation during storage^68^.

Previous studies of mated *Ae. aegypti* females have proposed that males transfer mRNA during mating—106 mRNAs have been identified as potentially male-derived^41,54^. We detected 6 of these transcripts in our differential expression analysis of spermathecal transcriptomes from NBF and BF females (File S6). Some mRNAs identified in the lower reproductive tract of mated females are derived from the male accessory glands^41,72^. Further, mammalian^73,74^ and *Drosophila*^75^ sperm contain a “pool” of RNAs. While sperm-specific RNAs have not been identified in *Ae. aegypti*, we wished to determine if the transcripts identified in our analysis might have originated from the testis or the accessory gland of the male reproductive tract. We compared the significantly up-regulated transcripts identified in the NBF and BF female groups to the male accessory gland and testis transcriptomes^72^. We found 11 transcripts that overlap with the testis transcriptome (0.56% of the total transcripts) and 36 that overlap with the accessory gland transcriptome (1.84%) (File S6). Of these genes, only one, the predicted head-peptide (AAEL024630), had no signal in the spermathecae of virgin females. However, we cannot rule out that the other 46 transcripts identified in the testes and/or accessory gland transcriptomes may have contributed to the signal we detected from the spermathecae of mated females. Further experimentation is required to determine the sex-specific expression of the testes/accessory gland transcripts also identified in our analysis.

### Functional categories of differentially expressed transcripts

We examined the major functional categories represented in the differentially expressed spermathecal genes from NBF and BF females compared to virgin females for all combined time points. Significant differences in GO terms were observed in up- and down-regulated transcripts between the NBF and BF groups. In the up-regulated dataset, we found 48 significantly overrepresented GO terms (FDR < 0.05) in the NBF group (File S7), with the majority related to transport activity. Other terms are associated with ATP activity, pH regulation and peptidases (File S7). The most significant terms correspond to energy-coupled proton transmembrane transport against an electrochemical gradient, ATP hydrolysis-coupled proton transport, proton transmembrane transport, ATPase activity and active transmembrane transporter activity (Table 1). In the BF group, we found 24 overrepresented GO terms related to amino acid metabolic processes, carboxylic acid metabolism, inosine monophosphate (IMP) metabolism and biosynthesis, transport activity and peptidase activity (File S7). The most significant terms corresponded to 'de novo' IMP biosynthetic process, integral component of membrane, secondary active transmembrane transporter activity, IMP biosynthetic process and IMP metabolic process (Table 1).

**Table 1:** The five most significant overrepresented GO terms (FDR < 0.05) for the differentially abundant spermathecal genes from NBF and BF females in the entire dataset.

Female status	Differential expression	Category	GO term	Ontology	FDR	Number of Genes
NBF	Upregulated	GO:0015988	Energy coupled proton transmembrane transport, against electrochemical gradient	BP	8.87E−13	13
NBF	Upregulated	GO:0015991	ATP hydrolysis coupled proton transport	BP	8.87E−13	13
NBF	Upregulated	GO:1902600	Proton transmembrane transport	BP	1.39E−09	13
NBF	Upregulated	GO:0046961	Proton-transporting ATPase activity, rotational mechanism	MF	3.56E−09	9
NBF	Upregulated	GO:0022804	Active transmembrane transporter activity	MF	3.90E−09	30
NBF	Downregulated	GO:0016021	Integral component of membrane	CC	0.003052399	114
NBF	Downregulated	GO:0031224	Intrinsic component of membrane	CC	0.004884063	116
NBF	Downregulated	GO:0005788	Endoplasmic reticulum lumen	CC	0.005784708	11
NBF	Downregulated	GO:0044432	Endoplasmic reticulum part	CC	0.005784708	42
NBF	Downregulated	GO:0008238	Exopeptidase activity	MF	0.009365785	16
NBF	Downregulated	GO:0019203	Carbohydrate phosphatase activity	MF	0.011186728	4
BF	Upregulated	GO:0006189	'De novo' IMP biosynthetic process	BP	0.002849721	5
BF	Upregulated	GO:0016021	Integral component of membrane	CC	0.002849721	110
BF	Upregulated	GO:0015291	Secondary active transmembrane transporter activity	MF	0.002849721	20
BF	Upregulated	GO:0006188	IMP biosynthetic process	BP	0.002849721	5
BF	Upregulated	GO:0046040	IMP metabolic process	BP	0.002849721	5
BF	Downregulated	GO:0003735	Structural constituent of ribosome	MF	8.38E−40	75
BF	Downregulated	GO:0005840	Ribosome	CC	1.17E−37	67
BF	Downregulated	GO:0006412	Translation	BP	2.72E−36	75
BF	Downregulated	GO:0044391	Ribosomal subunit	CC	1.27E−21	44
BF	Downregulated	GO:0022625	Cytosolic large ribosomal subunit	CC	8.04E−20	27

BP, biological process; MF, molecular function; CC, cellular component.

In the down-regulated dataset, we found 14 and 58 significantly overrepresented GO terms (FDR < 0.05) for the NBF and BF groups, respectively. Enriched terms in the NBF group are related to membrane components, endoplasmic reticulum, peptidases, proteasome core complex, cell redox homeostasis and proteolysis (File S7). In the BF group, there was a significant down regulation of several functions (File S7), with the most significant terms corresponding to structural constituent of ribosome, ribosome, translation, ribosomal subunit and cytosolic large ribosomal subunit (Table 1).

We next performed GO enrichment analysis of differentially expressed transcripts from NBF and BF female groups compared to virgin females at each post-mating time-point. In the up-regulated dataset at 6 h, significant functions are only observed in the BF group (Table 2; File S8), with the most significant terms corresponding to IMP biosynthetic process, IMP metabolic process, oxoacid metabolic process and carboxylic acid metabolic process (Table 2). At 24 h, we found 83 and 26 significant terms for the NBF and BF female groups, respectively. Overrepresented terms in the NBF group are related to transport activity and ATP transport and metabolism (File S8). The most significant terms are energy-coupled proton transmembrane transport and ATP hydrolysis coupled proton transport (Table 2). In the BF group, several terms are related with transmembrane transporter activity and ion transport (Table 2; File S8).

**Table 2:** The five most significant overrepresented GO terms (FDR < 0.05) for the differentially abundant spermathecal genes from NBF and BF females at each post-mating time point assessed.

Gene ontology analysis per time point						
Female status	Differential expression	Category	GO term	ontology	FDR	Number of Genes
NBF_24hrs	Upregulated	GO:0015988	Energy coupled proton transmembrane transport, against electrochemical gradient	BP	2.52E−15	13
NBF_24hrs	Upregulated	GO:0015991	ATP hydrolysis coupled proton transport	BP	2.52E−15	13
NBF_24hrs	Upregulated	GO:1902600	Proton transmembrane transport	BP	6.52E−12	13
NBF_24hrs	Upregulated	GO:0046961	Proton-transporting ATPase activity, rotational mechanism	MF	7.62E−11	9
NBF_24hrs	Upregulated	GO:0036442	Proton-exporting ATPase activity	MF	1.30E−10	9
NBF_72hrs	Upregulated	GO:0015291	Secondary active transmembrane transporter activity	MF	0.00068534	11
NBF_72hrs	Upregulated	GO:0055085	Transmembrane transport	BP	0.01174016	18
NBF_72hrs	Upregulated	GO:0015293	Symporter activity	MF	0.01174016	8
NBF_72hrs	Upregulated	GO:0022804	Active transmembrane transporter activity	MF	0.024988801	11
NBF_72hrs	Upregulated	GO:0015103	Inorganic anion transmembrane transporter activity	MF	0.04703175	6
NBF_24hrs	Downregulated	GO:0005788	Endoplasmic reticulum lumen	CC	0.003358554	8
NBF_24hrs	Downregulated	GO:0034663	Endoplasmic reticulum chaperone complex	CC	0.030130715	3
NBF_24hrs	Downregulated	GO:0005783	Endoplasmic reticulum	CC	0.030130715	15
NBF_24hrs	Downregulated	GO:0044432	Endoplasmic reticulum part	CC	0.030130715	20
NBF_24hrs	Downregulated	GO:0008238	Exopeptidase activity	MF	0.030130715	9
NBF_72hrs	Downregulated	GO:0044432	Endoplasmic reticulum part	CC	2.16E−10	36
NBF_72hrs	Downregulated	GO:0008233	Peptidase activity	MF	5.94E−09	35
NBF_72hrs	Downregulated	GO:0006508	Proteolysis	BP	3.11E−08	35
NBF_72hrs	Downregulated	GO:0070011	Peptidase activity, acting on L-amino acid peptides	MF	1.69E−07	32
NBF_72hrs	Downregulated	GO:0008238	Exopeptidase activity	MF	4.34E−07	15
BF_6hrs	Upregulated	GO:0006189	'De novo' IMP biosynthetic process	BP	8.92E−05	5
BF_6hrs	Upregulated	GO:0006188	IMP biosynthetic process	BP	8.92E−05	5
BF_6hrs	Upregulated	GO:0046040	IMP metabolic process	BP	8.92E−05	5
BF_6hrs	Upregulated	GO:0043436	Oxoacid metabolic process	BP	0.002182958	23
BF_6hrs	Upregulated	GO:0019752	Carboxylic acid metabolic process	BP	0.002182958	22
BF_24hrs	Upregulated	GO:0015291	Secondary active transmembrane transporter activity	MF	0.000542213	16
BF_24hrs	Upregulated	GO:0008514	Organic anion transmembrane transporter activity	MF	0.000555055	13
BF_24hrs	Upregulated	GO:0015711	Organic anion transport	BP	0.000555055	16
BF_24hrs	Upregulated	GO:0031224	Intrinsic component of membrane	CC	0.000555055	69
BF_24hrs	Upregulated	GO:0022804	Active transmembrane transporter activity	MF	0.000558125	19
BF_72hrs	Upregulated	GO:0015291	Secondary active transmembrane transporter activity	MF	0.016797097	13
BF_72hrs	Upregulated	GO:0044449	Contractile fiber part	CC	0.016797097	12
BF_72hrs	Upregulated	GO:0055085	Transmembrane transport	BP	0.047633823	25
BF_24hrs	Downregulated	GO:0008135	Translation factor activity, RNA binding	MF	2.40E−05	13
BF_24hrs	Downregulated	GO:0033180	Proton-transporting V-type ATPase, V1 domain	CC	9.60E−05	6
BF_24hrs	Downregulated	GO:0003735	Structural constituent of ribosome	MF	9.60E−05	18
BF_24hrs	Downregulated	GO:0006412	Translation	BP	0.000658026	18
BF_24hrs	Downregulated	GO:0003743	Translation initiation factor activity	MF	0.000672218	9
BF_72hrs	Downregulated	GO:0005840	Ribosome	CC	5.22E−49	67
BF_72hrs	Downregulated	GO:0003735	Structural constituent of ribosome	MF	5.04E−48	70
BF_72hrs	Downregulated	GO:0006412	Translation	BP	9.07E−46	70
BF_72hrs	Downregulated	GO:0019538	Protein metabolic process	BP	1.50E−33	139
BF_72hrs	Downregulated	GO:0005198	Structural molecule activity	MF	5.92E−27	74

BP, biological process; MF, molecular function; CC, cellular component.

Analysis of down-regulated transcripts showed statistically significant terms only at 24 and 72 h in both NBF and BF female groups (File S8). In NBF females, significant terms are related with endoplasmic reticulum and peptidases at 24 h (Table 2). An increase in the number of significant terms was observed at 72 h (File S8), with several related to proteolysis (Table 2). In the BF group, the most significant terms at 24 h were RNA binding proton-transporting V-type ATPase, and translation activity (Table 2). Significant GO terms increased at 72 h, related to ribosome, translation, protein metabolic process and structural molecule activity (Table 2).

### Comparative expression patterns between the spermathecae and lower reproductive tract tissues

The *Ae. aegypti* lower reproductive tract comprises the bursa, oviduct, spermathecal vestibule, spermathecal ducts and the spermathecae^21,40^. To determine transcripts more abundant in the spermathecae compared to the other reproductive tract tissues, we generated an overview of transcripts identified in this study compared to transcripts identified in the lower reproductive tract by Alfonso-Parra et al.^41^. We developed a comparative analysis to identify differentially expressed spermathecal transcripts from NBF females with the differentially expressed transcripts identified in all lower reproductive tract tissues at 6 and 24 h post-mating.

**Figure 5:**
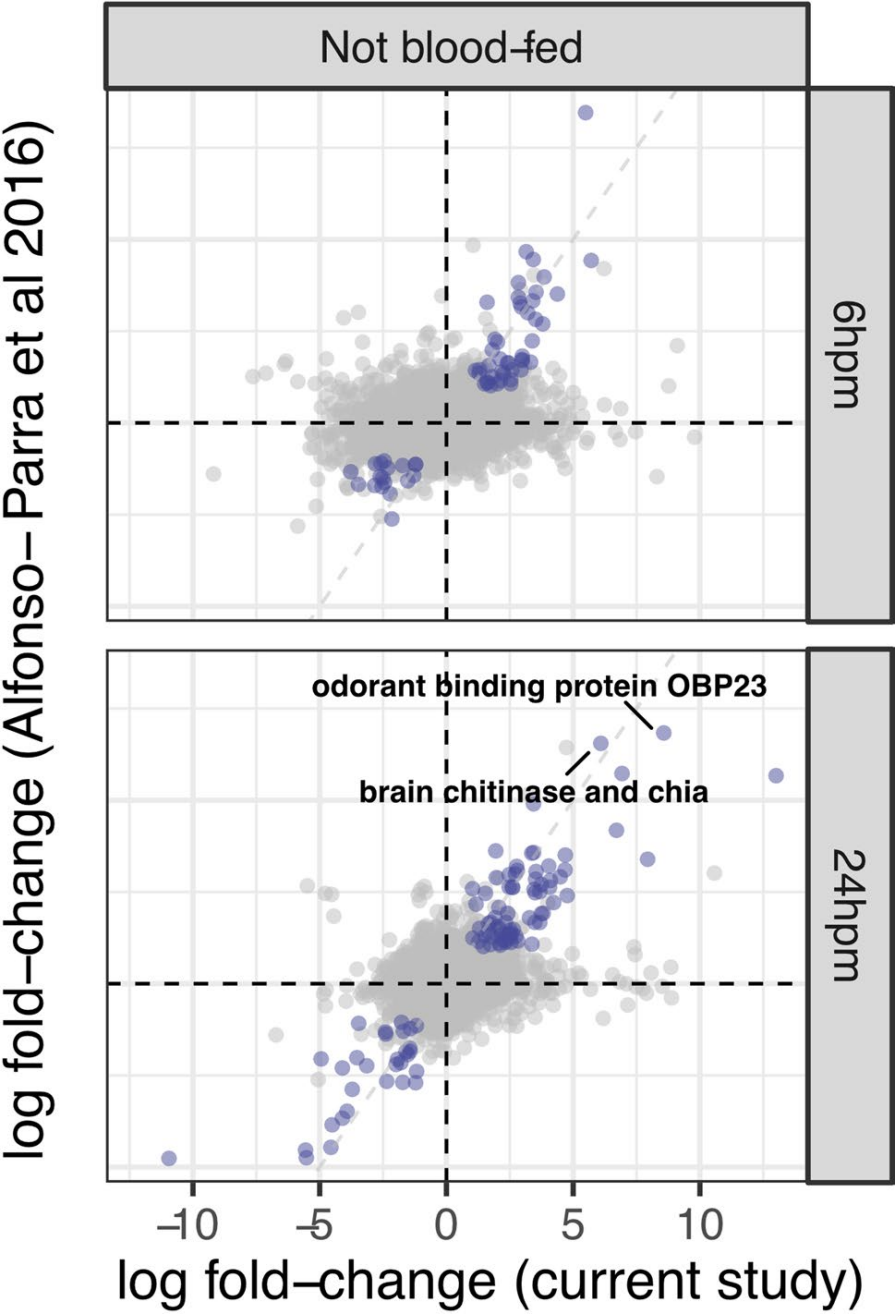
Comparative plot of the logFC for the 6 and 24 h post-mating in both spermathecae and lower reproductive tract tissues. Purple dots represent the differentially expressed genes in both studies at FDR < 0.05. Values below zero represent down-regulated genes and values above zero up-regulated genes.

A total of 116 differentially up-regulated transcripts were identified in both the NBF and BF datasets: 44 and 72 transcripts at 6 and 24 h, respectively (Fig. 5; File S9). The most significant up-regulated transcripts correspond to tyrosine-protein kinase hopscotch (AAEL003619) of the JAK/STAT signaling pathway at 6 h, odorant binding protein OBP23 (AAEL006109), and brain chitinase and chia (AAEL002972) at 24 h (Fig. 5; File S9). A total of 48 transcripts (18 at 6 h and 30 at 24 h) were found to be significantly down regulated in both studies (Fig. 5; File S9). The most significant down-regulated transcripts correspond to adenylate cyclase type 8 (AAEL022948) at 6 h and farnesol dehydrogenase-like (AAEL009685) at 24 h. While this comparison identified several overlapping genes, the number of unique transcripts found in the former study^41^ suggests that individual tissues of the *Ae. aegypti* female reproductive tract respond to mating. As mating induces changes in gene expression in reproductive tract tissues in addition to the spermathecae in other insects^69^, further analyses of *Ae. aegypti* female reproductive tract might uncover tissue-specific genes that are important in reproduction of this disease vector.

### Comparison of the early and late mated female spermathecae transcriptome

A recent study by Pascini et al.^31^ reported the spermathecae transcriptome in mated females at 7 d post-eclosion. We compared the differentially expressed genes in our datasets with those from the previous study (File S10), as these transcripts might represent long-term transcriptional changes in the spermathecae in response to mating. Seven days post-eclosion corresponds to ~ 6 d post-mating, as females require 24 h to reach sexual maturity and become receptive to mating^22^. Comparing the overall transcriptional response of the spermathecae in NBF females at 6, 24 and 72 h with that at 7 d post-eclosion, we found higher differentially expressed transcripts at each timepoint compared to the 7 d post-eclosion timepoint from Pascini et al.^31^. We also observed an overall decrease in gene expression levels in the NBF female group from 6 to 72 h, suggesting that the spermathecal transcriptional response decreases over time after mating. Moreover, 17 transcripts up-regulated at 6, 24 and 72 h were observed to be down-regulated at 7 d post-eclosion (File S10). Four up-regulated transcripts observed at 6 h in the NBF female group were found to be up-regulated at 7 d post-eclosion (File S10), suggesting that these genes return to high expression levels in the spermathecae nearly a week after mating.

Pascini et al.^31^ performed functional analysis on 8 genes found to be differentially regulated at 7 d post-eclosion. Of these 8 genes, we found only one in our dataset: the putative potassium-dependent sodium-calcium exchanger AAEL004814. In NBF female spermathecae, AAEL004814 transcripts are significantly up-regulated at 72 h post-mating. However, AAEL004814 had a greater response to blood-feeding: AAEL004814 transcripts were significantly down-regulated at 6 h, and significantly up-regulated at 24 and 72 h in the BF female group (File S4). The regulation of AAEL004814 by blood-ingestion is consistent with its role in oocyte development^31^.

## Conclusions

Immediately after mating ends, female insects undergo physiological and behavioral changes necessary for the production of progeny^15,16^. While several post-mating changes in female insects are stimulated after receipt of male seminal proteins^15^, spermathecal products are also required for optimal fertility^24,27,35^, suggesting that male and female-derived molecules might coordinate molecular activities to maximize reproductive success. Post-mating modification of female reproductive tract tissue^61^ or sperm in storage^72^ occur within hours of mating, and some seminal proteins that mediate sperm storage or ovulation require female contributions for optimal activity^76^ or undergo proteolytic cleavage in the sperm storage organs that are necessary to elicit their effects^26,77^. Mating responsive genes may be required for post-mating tissue or sperm modifications to occur and/or to mediate the effects of seminal proteins within the female reproductive tract.

The identification of reproductive molecules that are required for fertility is an important endeavor in developing desperately needed new strategies for vector control. Elucidating changes in mRNA expression after mating and blood-feeding in female *Ae. aegypti* spermathecae can identify genes that are important for sperm storage, and that thus can serve to monitor female mating physiology or provide novel molecular targets to control this important vector in nature. Unlike previous transcriptome studies in *Ae. aegypti* reproductive tissues that identified differentially expressed genes in mated, sugar-fed females, this study examined transcriptional change in response to both mating and blood-feeding in the spermathecae. We demonstrate that the spermathecal transcriptional response to mating affects regulation of transcripts related to osmotic balance, membrane transport and oxidoreduction. Furthermore, blood-feeding induces greater transcriptional change in the spermathecae compared to mating alone. Our results reveal the complexity of gene regulation in the *Ae. aegypti* spermathecae in response to mating and a blood-meal in the immediate days after mating when females undergo physiological and behavioral changes that are necessary for the production of progeny.

## Methods

### Mosquitoes

Thai strain *Ae. aegypti* was collected in Bangkok, Thailand and has been maintained in colony since 2009. Larval rearing and adult maintenance occurred in an environmental chamber at 27 °C, 80% relative humidity, and a 12 h dark: 12 h light photoperiod. Eggs were hatched under vacuum pressure (− 50 kPa) for 30 min in 250 ml dH_2_O supplemented with a pinch of active yeast. Resulting larvae were allocated to rearing trays 24 h later at a density of 200/1 L dH_2_O and fed with four Hikari Gold Cichlid Large Pellets (7.2–8.2 mm; Hikari, Himeju, Japan). Pupae were separated into individual 5 ml vials to ensure virginity. Adults were transferred to single sex 8 L cages until experiments commenced. Adults had constant access to 10% sucrose. Four to six-day-old adults were used in our experiments.

### Mating and spermathecae collection

We observed all matings by placing one female and three males into an 8 L bucket until a copulation occurred (defined as the engagement of male–female genitalia for ≥ 10 s^19,78^). The pair were subsequently removed, and the male discarded. Females were grouped into 20 min mating intervals. A subset of females were blood-fed on the arm of a volunteer immediately following a mating interval; only fully engorged females were considered blood-fed. Subsequent to mating and blood-feeding, females were housed in 500 ml cups and maintained in the environmental chamber until spermathecal dissection. Blood-feeding was approved by the Comité de Bioética Sede de Investigación Universitaria (Universidad de Antioquia); informed consent was obtained from all volunteers who participated in the study. All methods were performed in accordance with the relevant guidelines and regulations.

Spermathecal samples (from 3 biological replicates) were taken from (1) virgin females, (2) mated females and (3) mated + blood-fed females at 6, 24, and 72 h post-mating (Fig. 1). Females were anesthetized on ice and spermathecae dissected in sterile, 1X phosphate-buffered saline. Fat bodies and other associated tissues were carefully removed. The sample corresponded to a pool of 25 spermathecae (three complete reservoirs and ducts). Dissected spermathecae were placed into 100 μl QIAzol reagent (Qiagen, Hilden, Germany), centrifuged at maximum speed for 1 min and stored at − 80 **°C** until mRNA extraction.

### RNA extraction and library preparation

RNA extraction was based on a chloroform/isopropanol isolation and ethanol precipitation protocol following manufacturer’s instructions. GlycoBlue (Thermo Fisher Scientific, Waltham, USA) was added as a co-precipitant prior to ethanol precipitation. RNA concentration was measured using a Qubit spectrophotometer (Invitrogen, Grand Island, NY). At least 10 ng total RNA was used as input for polyA enrichment of mRNA. cDNA libraries were prepared using the NEBNext Ultra II Directional RNA Library Preparation kit for Illumina sequencing following the protocol for polyA enrichment with the NEBNext Poly(A) mRNA Magnetic Isolation kit (New England Biolabs, Ipswich, MA). Library preparation was performed at the Cornell University Transcriptional Regulation and Expression facility (Ithaca, NY).

### Sequencing and data analysis

Samples were sequenced using the NextSeq500 platform with 85nt single end reads at the Cornell Biotechnology Resource Center (Ithaca, NY). A minimum 20 M raw reads were generated per sample. Reads were processed using trim-galore (Babraham Institute) to filter low quality reads, filter noisy short fragment, and filter adapter sequences. Filtered reads were then aligned to the *Ae. aegypti* NCBI genome release (AaegL5.0) with RefSeq annotations using STAR^79^. A matrix of raw read counts for each annotated genomic feature was extracted. Genes with low read counts were filtered from the matrix (CPM ≤ 5), and RUVseq^80^ was used to identify sources of erroneous variation using residuals (k = 3), which were included in a design matrix that was entered into edgeR^81^ for differential abundance testing. Filtered reads were then aligned to the *Ae. aegypti* NCBI genome release (AaegL5.0) with RefSeq annotations using STAR. A matrix of raw read counts for each annotated genomic feature was extracted. Upon sample QC, we found that one replicate for virgin and one for the 24 h time point in BF females contained low quality data and were thus removed from downstream analysis. A total of 8,560 transcripts passed our quality control filters: 8,223 annotated and 337 unannotated transcripts on the VectorBase bioinformatics database (https://www.vectorbase.org). To perform gene-wise differential testing, we used a quasi-likelihood model and fit generalized linear models, and ultimately performed F-test to identify differentially abundant genes. To perform Gene Ontology (GO) enrichment analysis, we used ontology terms that were generated by Degner et al*.*^15^, and performed enrichment tests using the GOseq^82^ package in R. The R analysis script is available with this manuscript as Supplementary File S11.

## Supplementary information

Supplementary file1

Supplementary file2

Supplementary file3

Supplementary file4

Supplementary file5

Supplementary file6

Supplementary file7

Supplementary file8

Supplementary file9

Supplementary file10

## Data Availability

Data are available on NCBI under Accession Number PRJNA612326.
