# Supplementary material for: Mating and blood-feeding induce transcriptome changes in the spermathecae of the yellow fever mosquito Aedes aegypti
Source: Sci Rep. 2020 Sep 10;10:14899. doi: 10.1038/s41598-020-71904-z (PMC7484758; doi:10.1038/s41598-020-71904-z)
Supplement: Supplementary file 1 — Supplementary file1 [file 41598_2020_71904_MOESM1_ESM.pdf]

SUPPLEMENTARY INFORMATION

Mating and blood-feeding induce transcriptome changes in the spermathecae of the yellow fever mosquito *Aedes aegypti.*

Carolina Camargo[1], Yasir H. Ahmed-Braimah[2], I. Alexandra Amaro[3], Laura C. Harrington[3], Mariana F. Wolfner[4] and Frank W. Avila[1*]

1. Max Planck Tandem Group in Mosquito Reproductive Biology, Universidad de Antioquia, Medellín 050010, Colombia
2. Department of Biology, Syracuse University, Syracuse, NY 13244, United States
3. Department of Entomology, Cornell University, Ithaca, NY 14850, United States
4. Department of Molecular Biology and Genetics, Cornell University, Ithaca, NY 14850, United States

Figure S1. Normalization of transcripts before differential expression analysis **A.** Biological Coefficient Variation (BCV) mean-variance trend and model fit. **B.** Voom precision weights for mean variance trends. **C.** Quantile-Quantile (Q-Q plot) for the distribution of the sample transcript expression **D.** Log_2 counts per million trends per sample using voom transformed log counts per million (logCPM).

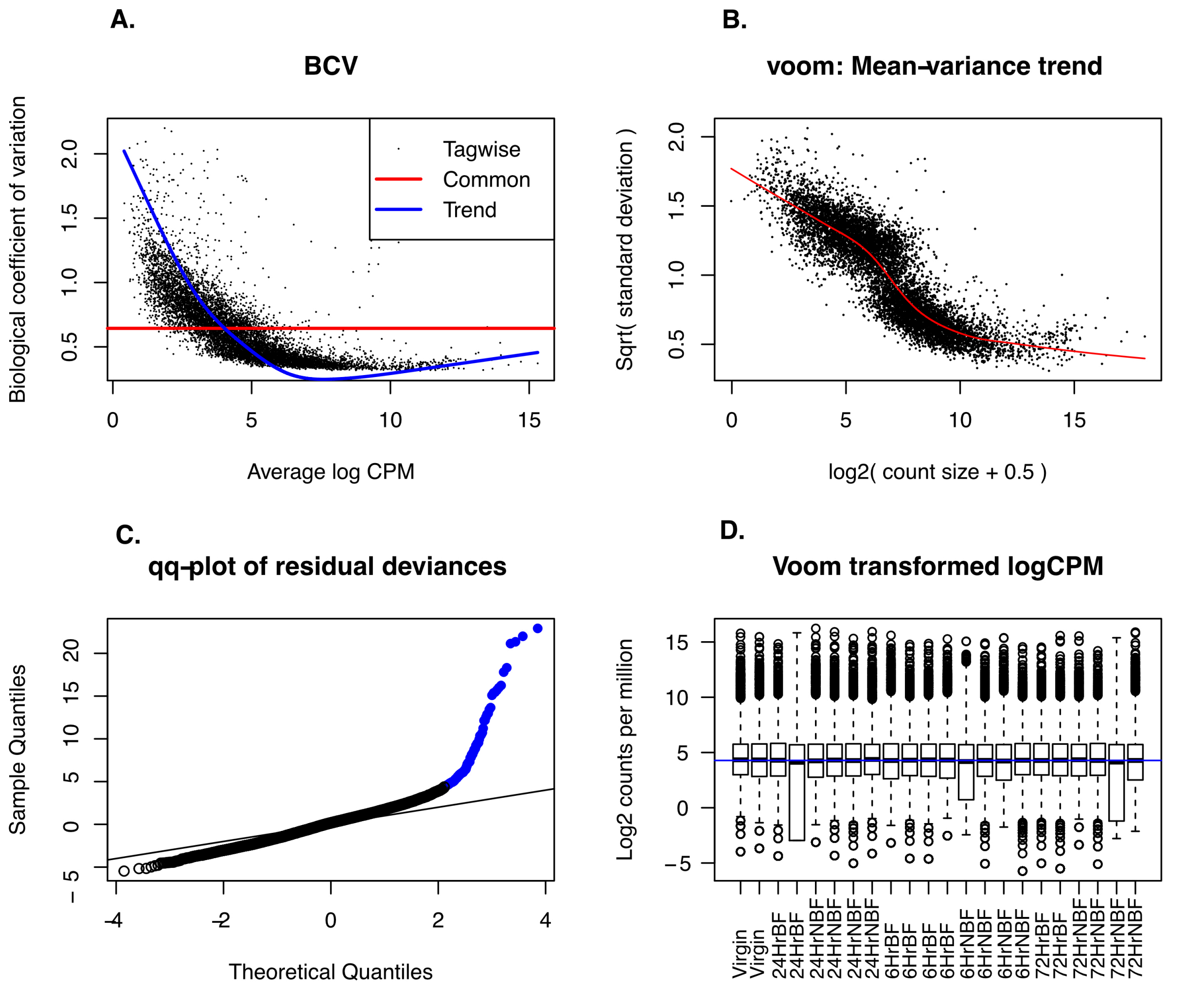

Figure S2. Quantile-Quantile (Q-Q plot) for the distribution of the transcript expression signal to noise ratio for all the comparisons made in the differential expression analysis. The dashed line shows the 95% normal distribution confidence intervals. The red dots show the transcripts cross the 95% confidence interval and were considered differentially expressed.

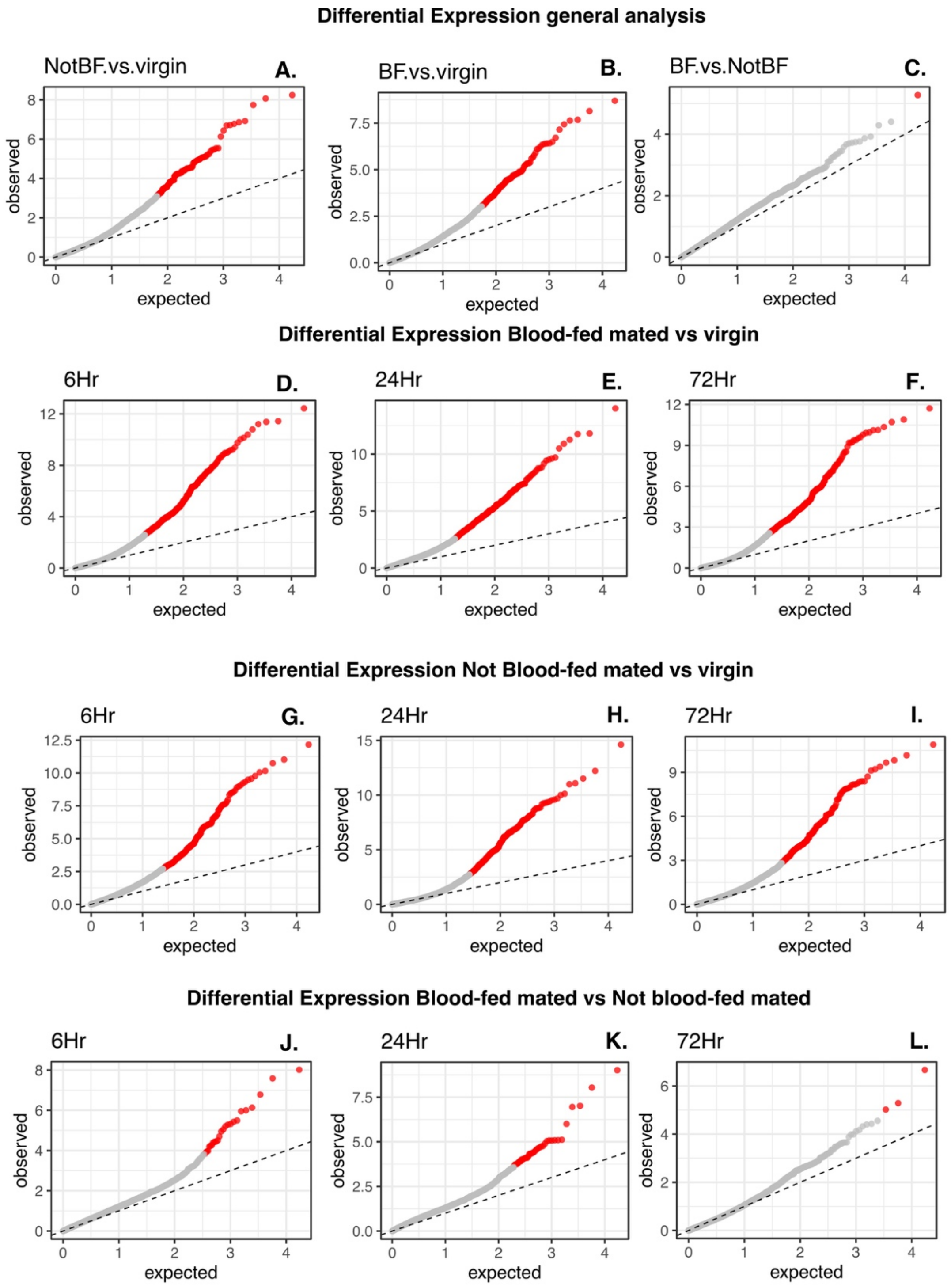

File S3. Differentially expressed transcripts in spermathecae from BF and NBF females compared to virgin females in the overall analysis.

File S4. Differentially expressed transcripts in spermathecae from NBF and BF females compared to virgin females at each post-mating timepoint.

File S5. Differentially expressed transcripts in spermathecae from BF females compared to NBF females at each post-mating timepoint.

File S6. Putative male-transferred transcripts identified in our analysis.

File S7. GO terms for the differentially expressed spermathecal genes of NBF and BF females compared to virgin females in the overall analysis.

File S8. GO terms differentially expressed spermathecal genes of NBF and BF females compared to virgin females at each post-mating timepoint.

File S9. Differentially up-regulated transcripts in both lower reproductive tract tissues (identified in Alfonso-Parra *et al.* 2016) and the spermathecae (identified in this analysis).

File S10. Comparison to Pascini et al. (2020).

File S11. R Script developed for our analysis.
