# Supplementary material for: Mating and blood-feeding induce transcriptome changes in the spermathecae of the yellow fever mosquito Aedes aegypti
Source: Sci Rep. 2020 Sep 10;10:14899. doi: 10.1038/s41598-020-71904-z (PMC7484758; doi:10.1038/s41598-020-71904-z)
Supplement: Supplementary file 5 — Supplementary file5 [file 41598_2020_71904_MOESM5_ESM.pdf]

File S6. Transcripts from the *Ae. aegypti* testes, male accessory gland (MAG) and mRNAs previously identified as male-transferred identified in the spermathecae transcriptome.

Transcripts differentially expressed from the testes (LogFC>1, FDR<0.05) were obtained from Degner et al. (2019) where they performed a likelihood ratio test on the GLM fit object that compares testes samples to gonadectomized carcass samples, MAG samples, and virgin female lower reproductive tract (LRT) samples. For MAGs, the same test was performed to compare carcass, testes and virgin female LRT to identify differentially expressed transcripts (LogFC>1, FDR<0.05) in those samples. Putative male-transferred mRNAs were identified by comparing LRTs from virgin and mated females at the 0 h timepoint (i.e. flash frozen immediately after genitalia disengagement). Each list was compared with our differentially up-regulated transcripts in the combined dataset and at each post-mating timepoint (6, 24 and 72 h).

Intersect of mRNAs expressed in the spermathecae and testes

A total of 11 transcripts from testes were identified in the upregulated differentially expressed transcripts in the spermathecae post-mating.

Intersect of testes vs spermathecae from BF females at 6 h
6 transcripts: "AAEL001052" "AAEL024717" "AAEL006642" "AAEL004738" "AAEL003581" "AAEL002377"

Intersect of testes vs spermathecae from NBF females at 6 h
2 transcripts: "AAEL006642" "AAEL024717"

Intersect of testes vs spermathecae from BF females at 24 h
2 transcripts: "AAEL024717" "AAEL013492"

Intersect of testes vs spermathecae from NBF females at 24 h
2 transcripts: "AAEL024717" "AAEL008789"

Intersect of testes vs spermathecae from BF females at 72 h
4 transcripts: "AAEL024717" "AAEL019441" "AAEL019844" "AAEL002543"

Intersect of testes vs spermathecae from NBF females at 72 h
2 transcripts: "AAEL019844" "AAEL002543"

Intersect of testes vs spermathecae from BF females timepoints at all timepoints combined
1 transcript: "AAEL024717"

Intersect of testes vs spermathecae from NBF females timepoints at all timepoints combined
1 transcript: "AAEL02471"

Intersect of mRNAs expressed in the spermathecae and the MAG.

A total of 36 transcripts from the MAG were identified in the up-regulated differentially expressed transcripts in the spermathecae post-mating.

Intersect of MAG vs spermathecae from BF females at 6 h
13 transcripts: "AAEL007191" "AAEL005676" "AAEL024098" "AAEL008039" "AAEL014053" "AAEL003606" "AAEL010777" "AAEL010727" "AAEL001134" "AAEL004193" "AAEL008473" "AAEL024630" "AAEL014526"

Intersect of MAG vs spermathecae from NBF females at 6 h
9 transcripts: "AAEL005676" "AAEL007191" "AAEL024098" "AAEL014053" "AAEL008473" "AAEL007777" "AAEL008039" "AAEL019596" "AAEL020035"

Intersect of MAG vs spermathecae from BF females at 24 h
12 transcripts: "AAEL007191" "AAEL011658" "AAEL014053" "AAEL008787" "AAEL008039" "AAEL004193" "AAEL014526" "AAEL002049" "AAEL009642" "AAEL012823" "AAEL015631" "AAEL019579"

Intersect of MAG vs spermathecae from NBF females at 24 h
18 transcripts: "AAEL011658" "AAEL014053" "AAEL007191" "AAEL002464" "AAEL008787" "AAEL012819" "AAEL012035" "AAEL009808" "AAEL002411" "AAEL012113" "AAEL007777" "AAEL005676" "AAEL008039" "AAEL003492" "AAEL005798" "AAEL010819" "AAEL002978" "AAEL011025"

Intersect of MAG vs spermathecae from BF females at 72 h
5 transcripts: "AAEL019579" "AAEL014053" "AAEL007191" "AAEL002049" "AAEL015631"

Intersect of MAG vs spermathecae from BF females at 72 h
5 transcripts: "AAEL019579" "AAEL007191" "AAEL020035" "AAEL025866" "AAEL024630"

Intersect of MAG vs spermathecae from BF females at all timepoints combined
4 transcripts: "AAEL007191" "AAEL015631" "AAEL004193" "AAEL011057"

Intersect of MAG vs spermathecae from NBF females at all timepoints combined
2 transcripts: "AAEL007191" "AAEL014053"

Intersect of mRNAs expressed in the spermathecae and potentially male transferred mRNA.

Previous studies of *Ae. aegypti* mated females identified 106 mRNAs that are potentially transferred from males to females during mating (Alfonso-Parra et al 2016; Degner et al. 2019). We identified 6 transcripts from these putative male transcripts in our upregulated differentially expressed transcripts in the spermathecae pots-mating.

Intersect of putative male mRNAs vs spermathecae from BF females at 6 h
2 transcripts: "AAEL007191" "AAEL024630"

Intersect of putative male mRNAs vs spermathecae from NBF females at 6 h
2 transcripts: "AAEL007191" "AAEL020035"

Intersect of putative male mRNAs vs spermathecae from BF females ta 24 h
1 transcript: "AAEL007191"

Intersect of putative male mRNAs vs spermathecae from NBF females at 24 h
3 transcripts: "AAEL007191" "AAEL003832" "AAEL003857"

Intersect of putative male mRNAs vs spermathecae from BF females at 72 h
1 transcript: "AAEL007191"

Intersect of putative male mRNAs vs spermathecae from NBF females at 72 h
4 transcripts: "AAEL007191" "AAEL020035" "AAEL025866" "AAEL024630"

Intersect of putative male mRNAs vs spermathecae from BF females at all timepoints combined
1 transcript: "AAEL007191"

Intersect of putative male mRNAs vs spermathecae from NBF females at all timepoints
1 transcript: "AAEL007191"
