# Supplementary material for: Mating and blood-feeding induce transcriptome changes in the spermathecae of the yellow fever mosquito Aedes aegypti
Source: Sci Rep. 2020 Sep 10;10:14899. doi: 10.1038/s41598-020-71904-z (PMC7484758; doi:10.1038/s41598-020-71904-z)
Supplement: Supplementary file 9 — Supplementary file9 [file 41598_2020_71904_MOESM9_ESM.pdf]

File S10. Comparison of the early (6, 24, and 72 h) and late (7 d post-eclosion) spermathecae transcriptomes

We identified differentially expressed transcripts in the spermathecae of NBF females at 6, 24, and 72 h post-mating that were also identified at 7 d post-eclosion reported in Pascini et. al (2020). To identify up- and down-regulated transcripts at 7 d post-eclosion, we used a range of Biological Coefficient variation (BCV) estimates to extract differentially expressed transcripts from the publicly available data from Pascini et. al (2020). We found that a BCV value of 0.1 gives results is comparable to our study in terms up- and down-regulated transcripts. This analysis was developed to have similarity in comparison with our transcriptomics data using a logFC approach. We identified 401 differentially expressed (DE) transcripts (294 up- and 107 down-regulated) in the mated female spermathecae at 7 d post-eclosion compared to virgins. After identifying DE transcripts at the 7 d post-eclosion timepoint we compared: **A.** up-regulated transcripts in our dataset (6, 24 and 72 h) and the 7 d post-eclosion timepoint, **B.** down-regulated transcripts in our dataset and the 7 d post-eclosion timepoint, and **C.** genes identified in our study with the 8 genes that underwent functional analysis in Pascini et al. (2020).

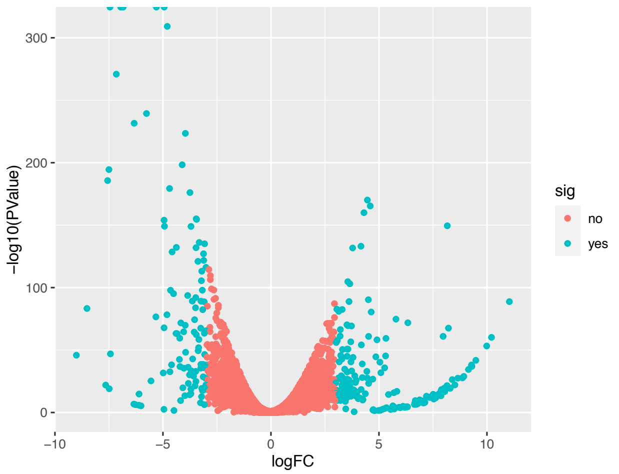

S10 Figure 1. Overview of transcriptional profile of up- and down-regulated (LogFC>1, FDR<0.05) genes at 7 d post-eclosion reported in Pascini et al. (2020).

A. Of the up-regulated transcripts identified in Pascini et al. (2020), we found the following four genes in our NBF female dataset:

1. LOC5576502: AAEL012553 – JAK/STAT pathway signaling Janus Kinase Hopscotch: Present at 6 h in NBF females.
2. LOC5570784: AAEL008567 – chymotrypsin-2: Present at 6 h in NBF females.
3. LOC5573696: AAEL010684 –trehalose-phosphate phosphatase: Present at 6 h NBF females.
4. LOC5570793: AAEL008564 – protein cueball: Present at 6 h NBF females.

B. Of the down-regulated transcripts identified in Pascini et al. (2020), we found the following genes in our NBF female dataset:

S10 Table 1. Transcripts down-regulated at 7 d post-eclosion identified in our NBF female datasets. Each transcript was found to up-regulated at the timepoint given.

NCBI_ID	VectorBase ID	GenBank_description	Timepoint
LOC5565484	AAEL004814	sodium/potassium/calcium exchanger 4	72h
LOC5575406	AAEL011809	glucose dehydrogenase [FAD%2C quinone] isoform X1	72h
LOC5577732	AAEL013367	mucin-5AC isoform X2	24h
LOC5566344	AAEL020936	carbonic anhydrase 1	24h
LOC5567429	AAEL006109	uncharacterized protein LOC5567429	24h
LOC23687933	AAEL017513	glycine-rich cell wall structural protein 1.0	6h
LOC5579095	AAEL003857	defensin-A-like	24h
LOC5571607	AAEL009198	major facilitator superfamily domain-containing protein 8 isoform X1	24h
LOC5574917	AAEL011522	ras-related and estrogen-regulated growth inhibitor-like protein	6h
LOC5579337	AAEL019957	gamma-glutamyltranspeptidase 1 isoform X2	24h
LOC5571084	AAEL008789	apolipophorin-3	24h
LOC5566873	AAEL000821	uncharacterized protein LOC5566873 isoform X1	24h
LOC110675563	AAEL023481	uncharacterized protein LOC110675563	24h
LOC5568181	AAEL023253	probable cationic amino acid transporter	24h
LOC5574115	AAEL002253	fringe glycosyltransferase	24h
LOC5575832	AAEL012103	aminopeptidase N isoform X1	72h
LOC5578811	AAEL013853	uncharacterized protein LOC5578811	72h
LOC5563917	AAEL003996	protein Teyrha-meyrha isoform X3	6h

C. Pascini et al. (2020) performed functional analysis of the following genes. We found only one gene in our datasets (AAEL004814):

Ae-92048 - AAEL009204: glucose dehydrogenase. (not found in our DE genes). Note: we identified one glucose dehydrogenase (AAEL011809) in NBF females: down-regulated at 6 h, up-regulated at 72 h post-mating; BF females: down-regulated at 6 h, up-regulated at 24 and 72 h.

Ae-187521 - AAEL002725 (not found in our DE genes).

Ae-27176 - AAEL005850: Atrophin-1 protein (not found in our DE genes).

AeSigP-4002 - AAEL007989: DHR4 ligand (not found in our DE genes).

Ae-88956 - AAEL029061: Chitin-binding type-2 (not found in our DE genes).

AeSigP-66427 - AAEL004814: Na^{+}/Ca^{2+} exchanger protein (not found in the overall dataset; found in BF females: 6 h DOWN, BF 24 h UP; 72 h UP; NBF females 72 h UP).

AeSigP-212177 - AAEL020068: N-acetylgalactosaminyl transferase 6 (not found in our DE genes).

AeSigP-109183 - Kazal-type serine protease inhibitor. No vectorbase ID available; we were unable to identify this gene in the *Ae. aegypti* NCBI genome release (AaegL5.0) given the nucleotide/amino acid sequences reported in Pascini et al. (2020).
