# Supplementary material for: Mating and blood-feeding induce transcriptome changes in the spermathecae of the yellow fever mosquito Aedes aegypti
Source: Sci Rep. 2020 Sep 10;10:14899. doi: 10.1038/s41598-020-71904-z (PMC7484758; doi:10.1038/s41598-020-71904-z)
Supplement: Supplementary file 10 — Supplementary file10 [file 41598_2020_71904_MOESM10_ESM.pdf]

In [ ]:

```
##Load the required R packages for analyses/plotting:

req_packages = c("data.table", "edgeR", "ggrepel", "reshape", "Rmisc", "goseq",
"qvalue", "GO.db", "RUVSeq", "tidyverse", "pheatmap", "ggpubr", "wesanderson",
"VennDiagram","grid", "gridExtra","cowplot", "viridis", "ggplot2")

invisible(suppressWarnings(suppressMessages(
  lapply(req_packages, require, character.only = TRUE)
)))

theme_set(theme_bw(base_size = 16))
```

In [ ]:

```
## suppress excessive VennDiagram log files
futile.logger::flog.threshold(futile.logger::ERROR, name = "VennDiagramLogger")

source("Functions.R")
```

In [ ]:

```
## Load Gene Counts
geneCounts = read.csv(file = "counts_matrix.txt", header = T, sep = "\t", row.na
mes = 1, check.names = F)
geneCounts = select(geneCounts, -`18_24HrBF`, -`15_Virgin`)
```

In [ ]:

```
##Load the comprehensive expression data:

compRNAseq = read.csv("aegypti_comp_RNAseq_data_v2.txt", header = T, sep = "\t")
compRNAseq
```

In [ ]:

```
## Load Data Lower Reproductive Tract

transferred.mRNAs <- as.character(read.table("transferred.mRNAs.txt", header = F
, sep = "\t")$V1)
transferred.mRNAs
```

In [ ]:

```
##Load sample/replicate information

sampleData = read.table("samples.txt", header=F, check.names=F)

colnames(sampleData) = c("Sample", "Replicate")
sampleData = filter(sampleData, Replicate != "18_24HrBF" & Replicate != "15_Virg
in")


###Add columns for factor groupings

sampleData$mating_status = ifelse(sampleData$Sample == "Virgin", "unmated", "mat
ed")

sampleData$BF_status = ifelse(grepl("NBF", sampleData$Sample), "NotBF", ifelse(s
ampleData$Sample == "Virgin", "virgin", "BF"))

sampleData$time = gsub(".*_", "", sampleData$Sample)


##Load genbank ID information:

gene_ids = read.csv("GenBank_IDs_description.txt", header = T, sep = "\t")
locID_to_aaelID = unique(subset(gene_ids, select = c("loc_ID", "active_id")))

###Some aditional annotations from previous studies

trinotate = read.csv("Trinotate_report.xls_with_description_columns.txt", header
= T, sep = "\t", na.strings = "", stringsAsFactors=FALSE)

AaegL5_annotation = read.csv("AaegL5.gene.annotation.txt", header = T, sep = "\t
")

annTrin = unique(select(trinotate, gene_id, sprot_Top_BLASTX_hit_description))

Annots = merge(AaegL5_annotation, annTrin, by.x = "gene_id", by.y = "gene_id", a
ll = T)
```

In [ ]:

```
############Mating alters the transcript profile in the spermathecae.###########
########
############Checking for Normality and Data Quality Control ############
###Make a TPM matrix, first make a tpm function:

# TPM function
tpm <- function(counts, lengths) {
    rate <- counts / lengths
    rate / sum(rate) * 1e6
  }

geneTPM <- tpm(geneCounts, 2977)

##Reshape the TPM matrix and add sample information:

tmp.tpmMatrix<-geneTPM
# colnames(tmp.tpmMatrix) <- sampleData$Sample
tmp.tpmMatrix.m <- as.data.frame(melt(as.matrix(tmp.tpmMatrix)))
colnames(tmp.tpmMatrix.m) <- c("gene_id", "replicate", "TPM")
geneTPM.table <- merge(tmp.tpmMatrix.m, sampleData, by.x = "replicate", by.y =
"Replicate", all.x = T)


##Check the library sizes:

a.bt2LibSizes <- as.data.frame(colSums(geneCounts))
libSizes = cbind(a.bt2LibSizes)
libSizes <- cbind(sample = row.names(libSizes), libSizes)
row.names(libSizes)<- NULL
colnames(libSizes) = c("sample", "genome")
libSizes = melt(libSizes, id.vars = "sample")
colnames(libSizes) = c("sample", "mapping_locale", "mapped_reads")

options(repr.plot.width = 9, repr.plot.height = 3.5)
# pdf("Figures/Library_sizes.pdf", width = 12, height = 4)
ggplot(libSizes, aes(sample, mapped_reads, fill = mapping_locale)) +
geom_bar(stat="identity", position = "dodge") +
#     theme_monokai_full() +
theme(axis.text.x = element_text(angle = 45, hjust = 1)) +
geom_hline(yintercept = 10000000, colour = "yellow", linetype= "dashed") +
scale_fill_manual(values = c("#ff8a5e","#02aded"))
# dev.off()


##First, we have to filter out genes with very low expression from the counts ma
trix. We'll use a minimum cutoff of 5 CPM in at least 3 samples:

gene.CPM <- cpm(geneCounts)
thresh <- gene.CPM > 5
keep <- rowSums(thresh) >= 3
counts.keep <- geneCounts[keep,]
dim(counts.keep)

##Set up a design matrix for DE analysis:

bf.groups = factor(sampleData$BF_status)
bf.design = model.matrix( ~ 0 + bf.groups)
colnames(bf.design) <- levels(bf.groups)
rownames(bf.design) <- sampleData$Replicate
```

```
##Make an expression set to input for RUVseq:

set <- newSeqExpressionSet(as.matrix(counts.keep), phenoData = data.frame(bf.gro
ups, row.names = colnames(counts.keep)))
set <- betweenLaneNormalization(set, which="upper")

##Now estimate the sources of uwanted variation using RUVseq:

y <- DGEList(counts=counts(set), group=bf.groups)
y <- calcNormFactors(y, method="upperquartile")
y <- estimateDisp(y, bf.design, robust = T)
fit <- glmQLFit(y, bf.design, dispersion = y$tagwise.dispersion, robust = T)
res <- residuals(fit, type="deviance")

##Use the residual to estimate the factors of unwanted variation:

batch_ruv_res = RUVr(set,rownames(counts.keep),k=3,res)
RUVrNormalizedCounts = normCounts(object = batch_ruv_res)
rownames(RUVrNormalizedCounts) = rownames(counts.keep)

##Make a new TPM matrix with adjusted counts:

geneTPM_adjusted <- apply(RUVrNormalizedCounts, 2, function(x) tpm(x, 2000))

tmp.tpmMatrix<-geneTPM_adjusted

# colnames(tmp.tpmMatrix) <- sampleData$Sample
tmp.tpmMatrix.m <- as.data.frame(melt(as.matrix(tmp.tpmMatrix)))
colnames(tmp.tpmMatrix.m) <- c("gene_id", "replicate", "TPM")
geneTPM_adjusted.table <- merge(tmp.tpmMatrix.m, sampleData, by.x = "replicate",
by.y = "Replicate", all.x = T)

###Set up a new design matrix with the unwanted factors specified:

bf.design_2 <- model.matrix(~ 0 + bf.groups + W_1 + W_2 + W_3, data=pData(batch_
ruv_res))
colnames(bf.design_2) <- gsub("bf.groups", "", colnames(bf.design_2))

##Check the correction values:

options(repr.plot.width = 5, repr.plot.height = 3)
plot(batch_ruv_res$W_1,pch=19,main="unsupervised sva")

##Create the DGElist object and GLM fit:
dgeList <- DGEList(counts = counts(set), group = bf.groups)
dgeList <- calcNormFactors(dgeList)
dgeList <- estimateGLMCommonDisp(dgeList, bf.design_2)
dgeList <- estimateGLMTagwiseDisp(dgeList, bf.design_2)
dgeList <- estimateGLMTrendedDisp(dgeList, bf.design_2)
dgeList_fit <- glmQLFit(dgeList, bf.design_2, dispersion = dgeList$trended.dispe
rsion)
summary(dgeList$tagwise.dispersion)
```

In [ ]:

```
############ Mating alters the transcript profile in the spermathecae ##########
#########

#####################################################
##Comparison between BF and non BF overall analysis##
#####################################################

###Look at BCF, mean-variance trend and model fit:
options(repr.plot.width = 9, repr.plot.height = 6)
par(mfrow=c(2,2))

# Biological coefficient of variation
plotBCV(dgeList, main = "BCV")

# mean-variance trend
voom = voom(dgeList, bf.design_2, plot=TRUE)

# model fit
gof(dgeList_fit, pcutoff = 0.05, adjust = "holm", plot = T)

# log2 transformed and normalize boxplot of counts across samples

boxplot(voom$E, xlab="", ylab="Log2 counts per million",las=2,main="Voom transfo
rmed logCPM")
abline(h=median(voom$E),col="blue")

## Plot sample correlation
data = log2(RUVrNormalizedCounts+1)
data = as.matrix(data)
sample_cor = cor(data, method='pearson', use='pairwise.complete.obs')

sampleColLabeling = sampleData
rownames(sampleColLabeling) = sampleColLabeling$Replicate
sampleColLabeling = subset(sampleColLabeling, select = c("mating_status", "BF_st
atus", "time"))

options(repr.plot.width = 8.5, repr.plot.height = 7)
pheatmap(
  mat                   = sample_cor,
  show_colnames         = TRUE,
  show_rownames         = TRUE,
  annotation_col        = sampleColLabeling,
  annotation_row        = sampleColLabeling,
  fontsize              = 12
)
rm(data)
rm(sample_cor)


##An MDS plot to see how samples are grouping:

x <- DGEList(counts = RUVrNormalizedCounts, group = bf.groups)
suppressWarnings(x <- calcNormFactors(x))
x <- estimateGLMCommonDisp(x, bf.design)
x <- estimateGLMTagwiseDisp(x, bf.design)
x <- estimateGLMTrendedDisp(x, bf.design)

mdsObj <- plotMDS(x, plot = F)$cmdscale.out
mdsObj <- as.data.frame(as.matrix(mdsObj))
```

```
mdsObj$replicate <- rownames(mdsObj)
colnames(mdsObj) = c("dim1", "dim2", "replicate")
mdsObj = merge(mdsObj, sampleData, by.x = "replicate", by.y = "Replicate")

options(repr.plot.width = 5, repr.plot.height = 3)
ggscatter(mdsObj,
          x = "dim1",
          y = "dim2",
          color = "time",
          shape = "BF_status",
          size = 3.5,
          alpha = 0.8,
          ggtheme = theme_bw(),
          repel = "time",
) +
  theme(axis.text = element_text(size = 10), legend.title = element_blank(), axi
s.title = element_text(size = 12), legend.text = element_text(size = 12)) +
  theme_bw() +
  labs ( x = "Dimension 1", y = "Dimension 2")

##Combine annotation information with the dgeList object:

# Extract annotation for genes in the fit object
ann = subset(gene_ids, loc_ID %in% rownames(dgeList_fit))
# convert factors to characters
ann = data.frame(lapply(ann, as.character), stringsAsFactors=FALSE)
# align the fit object's rownames with gene ID's
ann = ann[match(rownames(dgeList_fit), ann$loc_ID),]
# convert factors to characters, again
ann <- data.frame(lapply(ann, as.character), stringsAsFactors=FALSE)
# Rename "FBgn_ID" as "GeneID"
colnames(ann) = c ("GeneID", "VB_ID", "GenBank_description")
# Check that the fit rownames match the annotation file's gene ID's
table(ann$GeneID==rownames(dgeList_fit))
# Add the annotations to the fit object in the "genes" slot
dgeList_fit$genes = ann
```

In [ ]:

```
###########Mating alters the transcript profile in the spermathecae.###########
########

###Contrast of the Overall Analysis
##Now we'll run the contrasts between each postmating sample and the virgin samp
le:

BF.vs.virgin <- makeContrasts(BF-virgin, levels = bf.design_2)
NotBF.vs.virgin <- makeContrasts(NotBF-virgin, levels = bf.design_2)
BF.vs.NotBF <- makeContrasts(BF-NotBF, levels = bf.design_2)

##The blood-fed vs. virgin combined comparison:
Ftest.BF.vs.virgin <- glmQLFTest(dgeList_fit, contrast = BF.vs.virgin)
Ftest.BF.vs.virgin.tTags <- topTags(Ftest.BF.vs.virgin, n = NULL)
Ftest.BF.vs.virgin.tTags.table <- Ftest.BF.vs.virgin.tTags$table
Ftest.BF.vs.virgin.tTags.table$sig = ifelse(Ftest.BF.vs.virgin.tTags.table$FDR <
0.05 & (Ftest.BF.vs.virgin.tTags.table$logFC < -1 | Ftest.BF.vs.virgin.tTags.tab
le$logFC > 1), "yes", "no")
Ftest.BF.vs.virgin.tTags.table$comparison = "blood-fed and mated"
nrow(subset(Ftest.BF.vs.virgin.tTags.table, sig == "yes"  & (logFC > 1 | logFC <
-1)))

###Ftest.NBF.vs.virgin <- glmQLFTest(dgeList_fit, contrast = NotBF.vs.virgin)
Ftest.NBF.vs.virgin <- glmQLFTest(dgeList_fit, contrast = NotBF.vs.virgin)
Ftest.NBF.vs.virgin.tTags <- topTags(Ftest.NBF.vs.virgin, n = NULL)
Ftest.NBF.vs.virgin.tTags.table <- Ftest.NBF.vs.virgin.tTags$table
Ftest.NBF.vs.virgin.tTags.table$sig = ifelse(Ftest.NBF.vs.virgin.tTags.table$FDR
< 0.05 & (Ftest.NBF.vs.virgin.tTags.table$logFC < -1 | Ftest.NBF.vs.virgin.tTags
.table$logFC > 1), "yes", "no")
Ftest.NBF.vs.virgin.tTags.table$comparison = "not blood-fed and mated"
nrow(subset(Ftest.NBF.vs.virgin.tTags.table, sig == "yes" & (logFC > 1 | logFC <
-1)))

##The blood-fed vs. not blood-fed combined comparison:
Ftest.BF.vs.NBF <- glmQLFTest(dgeList_fit, contrast = BF.vs.NotBF)
Ftest.BF.vs.NBF.tTags <- topTags(Ftest.BF.vs.NBF, n = NULL)
Ftest.BF.vs.NBF.tTags.table <- Ftest.BF.vs.NBF.tTags$table
Ftest.BF.vs.NBF.tTags.table$sig = ifelse(Ftest.BF.vs.NBF.tTags.table$FDR < 0.05
& (Ftest.BF.vs.NBF.tTags.table$logFC < -1 | Ftest.BF.vs.NBF.tTags.table$logFC >
1), "yes", "no")
Ftest.BF.vs.NBF.tTags.table$comparison = "not blood-fed and mated"
nrow(subset(Ftest.BF.vs.NBF.tTags.table, sig == "yes" & (logFC > 1 | logFC < -1
)))

##Check the qq-plots:
options(repr.plot.width = 3, repr.plot.height = 2.2)
ggplot.qq(Ftest.BF.vs.virgin.tTags.table$PValue, hit.idx = which(Ftest.BF.vs.vir
gin.tTags.table$FDR < 0.05)) + ggtitle("BF.vs.virgin")
ggplot.qq(Ftest.NBF.vs.virgin.tTags.table$PValue, hit.idx = which(Ftest.NBF.vs.v
irgin.tTags.table$FDR < 0.05)) + ggtitle("NotBF.vs.virgin")
ggplot.qq(Ftest.BF.vs.NBF.tTags.table$PValue, hit.idx = which(Ftest.BF.vs.NBF.tT
ags.table$FDR < 0.05)) + ggtitle("BF.vs.NotBF")

head(Ftest.BF.vs.virgin.tTags.table)
```

In [ ]:

```
########### Figure 2 ###############
##Check the data of BF and NBF mated females vs virgin with a volcano plot:
dev.off()
Ftest.mated.tTags.table = rbind(Ftest.BF.vs.virgin.tTags.table, Ftest.NBF.vs.vir
gin.tTags.table)
head(Ftest.mated.tTags.table)
options(repr.plot.width = 7, repr.plot.height = 4)
ggplot(Ftest.mated.tTags.table, aes(logFC, -log10(PValue), colour = sig)) +
  geom_point(alpha = 0.7, size = 1) +
  facet_wrap(~comparison) +
  #     theme_monokai_full() +
  scale_colour_manual(values = c("grey", "#F8766D")) +
  geom_vline(xintercept = 0, linetype = "dashed", colour = "black") +
  geom_text_repel(data = subset(Ftest.mated.tTags.table, sig == "yes" & -log10(P
Value) > 7), aes(logFC, -log10(PValue),label=VB_ID),
                  force = 50,
                  inherit.aes = F,
                  box.padding = unit(0.35, "lines"),
                  point.padding = unit(0.5, "lines"),
                  fontface = "bold",
                  size = 3)
#ggtitle("DE between blod-fed and \nnonblood-fed mated females")
ggsave("BF.vs.virgin_VolcanoPlots2.pdf", width = 7, height = 3.5)

##################################
##Make a Venn diagram of upregulated and downregulated genes:
#################################

upregulated_candidates <- list(BF = subset(Ftest.BF.vs.virgin.tTags.table, sig =
= "yes" & logFC > 1 & !is.na(GeneID))$GeneID, NBF = subset(Ftest.NBF.vs.virgin.t
Tags.table, sig == "yes" & logFC > 1 & !is.na(GeneID))$GeneID)

downregulated_candidates <- list(BF = subset(Ftest.BF.vs.virgin.tTags.table, sig
== "yes" & logFC < -1 & !is.na(GeneID))$GeneID, NBF = subset(Ftest.NBF.vs.virgi
n.tTags.table, sig == "yes" & logFC < -1 & !is.na(GeneID))$GeneID)

upregulated_Vdiag<-venn.diagram(upregulated_candidates, NULL, fill=c("#F8766D",
"#0075e1"), alpha=c(0.75,0.75), cex = 1.5, cat.fontface= 6, cat.cex = 1, resolut
ion = 100, main = "upregulated", main.cex = 1, cat.dist = c(0.05, 0.05), main.di
st = -0.9)

downregulated_Vdiag<-venn.diagram(downregulated_candidates, NULL, fill=c("#F8766
D", "#0075e1"), alpha=c(0.75,0.75), cex = 1.5, cat.fontface= 6, cat.cex = 1, res
olution = 100, main = "downregulated", main.cex = 1, cat.dist = c(0.01, 0.05), m
ain.dist = -0.9)

options(repr.plot.width = 5, repr.plot.height =2)
grid.arrange(gTree(children=upregulated_Vdiag), gTree(children=downregulated_Vdi
ag), ncol = 2, top = "DE genes between mated and virgin RT samples")

##Make list objects for the genes included in these venn diagrams:

##Downregulated
downregulated_combs <- unlist(lapply(1:length(downregulated_candidates), functio
n(j) combn(names(downregulated_candidates), j, simplify = FALSE)), recursive = F
ALSE)

names(downregulated_combs) <- sapply(downregulated_combs, function(i) paste0(i,
collapse = ","))
```

```
downregulated_elements <- lapply(downregulated_combs, function(i) Setdiff(downre
gulated_candidates[i], downregulated_candidates[setdiff(names(downregulated_cand
idates), i)]))

summary(downregulated_elements)

##Upregulated
upregulated_combs <- unlist(lapply(1:length(upregulated_candidates), function(j)
combn(names(upregulated_candidates), j, simplify = FALSE)), recursive = FALSE)

names(upregulated_combs) <- sapply(upregulated_combs, function(i) paste0(i, coll
apse = ","))

upregulated_elements <- lapply(upregulated_combs, function(i) Setdiff(upregulate
d_candidates[i], upregulated_candidates[setdiff(names(upregulated_candidates), i
)]))

summary(upregulated_elements)

##Extract the genes from the Venn diagrams in the form of a table. Here's an exa
mple of how to: do that with the "upregulated_elements" object:

tmp.venn = as.data.frame(unlist(upregulated_elements))
tmp.venn$group = rownames(tmp.venn)
tmp.venn$group = gsub("[[:digit:]]", "", tmp.venn$group)
colnames(tmp.venn) = c("gene", "group")
head(tmp.venn)

write.table(tmp.venn, file="upregulated_elements.csv", sep = ",")

tmp.venn = as.data.frame(unlist(downregulated_elements))
tmp.venn$group = rownames(tmp.venn)
tmp.venn$group = gsub("[[:digit:]]", "", tmp.venn$group)
colnames(tmp.venn) = c("gene", "group")
head(tmp.venn)

write.table(tmp.venn, file="dowregulated_elements.csv", sep = ",")
```

In [ ]:

```
####################################################################
###  Temporal transcriptional profile dynamics of the spermathecae
###    in response to mating and blood-feeding ###################
####################################################################

##Post-mating contrasts with time-point and blood-feeding status
################################################################

##Set up a design matrix for DE analysis:
groups = factor(sampleData$Sample)
design = model.matrix( ~ 0 + groups)
colnames(design) <- levels(groups)
rownames(design) <- sampleData$Replicate


##Make an expression set to input for RUVseq:

set <- newSeqExpressionSet(as.matrix(counts.keep), phenoData = data.frame(groups
, row.names = colnames(counts.keep)))
set <- betweenLaneNormalization(set, which="upper")


##Now estimate the sources of uwanted variation using RUVseq:

y <- DGEList(counts=counts(set), group=groups)
y <- calcNormFactors(y, method="upperquartile")
y <- estimateDisp(y, design, robust = T)
fit <- glmQLFit(y, design, dispersion = y$tagwise.dispersion, robust = T)
res <- residuals(fit, type="deviance")

##Use the residual to estimate the factors of unwanted variation:

batch_ruv_res = RUVr(set,rownames(counts.keep),k=1,res)
RUVrNormalizedCounts = normCounts(object = batch_ruv_res)
rownames(RUVrNormalizedCounts) = rownames(counts.keep)

##Make a new TPM matrix with adjusted counts:

geneTPM_adjusted <- apply(RUVrNormalizedCounts, 2, function(x) tpm(x, 2000))
tmp.tpmMatrix<-geneTPM_adjusted

# colnames(tmp.tpmMatrix) <- sampleData$Sample
tmp.tpmMatrix.m <- as.data.frame(melt(as.matrix(tmp.tpmMatrix)))
colnames(tmp.tpmMatrix.m) <- c("gene_id", "replicate", "TPM")
geneTPM_adjusted.table <- merge(tmp.tpmMatrix.m, sampleData, by.x = "replicate",
by.y = "Replicate", all.x = T)

##Set up a new design matrix with the unwanted factors specified:

design_2 <- model.matrix(~ 0 + groups + W_1, data=pData(batch_ruv_res))
colnames(design_2) <- gsub("groups", "", colnames(design_2))
Check the correction values:

options(repr.plot.width = 5, repr.plot.height = 3)
plot(batch_ruv_res$W_1,pch=19,main="unsupervised sva")

##Create the DGElist object and GLM fit:

dgeList <- DGEList(counts = counts(set), group = groups)
```

```
dgeList <- calcNormFactors(dgeList)
dgeList <- estimateGLMCommonDisp(dgeList, design_2)
dgeList <- estimateGLMTagwiseDisp(dgeList, design_2)
dgeList <- estimateGLMTrendedDisp(dgeList, design_2)
dgeList_fit <- glmQLFit(dgeList, design_2, dispersion = dgeList$trended.dispersi
on)
summary(dgeList$tagwise.dispersion)

##Look at BCF, mean-variance trend and model fit:

options(repr.plot.width = 9, repr.plot.height = 6)
par(mfrow=c(2,2))

# Biological coefficient of variation
plotBCV(dgeList, main = "BCV")

# mean-variance trend
voom = voom(dgeList, design_2, plot=TRUE)

# model fit
gof(dgeList_fit, pcutoff = 0.05, adjust = "holm", plot = T)

# log2 transformed and normalize boxplot of counts across samples
boxplot(voom$E, xlab="", ylab="Log2 counts per million",las=2,main="Voom transfo
rmed logCPM")
abline(h=median(voom$E),col="blue")

########
##An MDS plot to see how samples are grouping:

x <- DGEList(counts = RUVrNormalizedCounts, group = groups)
suppressWarnings(x <- calcNormFactors(x))
x <- estimateGLMCommonDisp(x, design)
x <- estimateGLMTagwiseDisp(x, design)
x <- estimateGLMTrendedDisp(x, design)

mdsObj <- plotMDS(x, plot = F)$cmdscale.out
mdsObj <- as.data.frame(as.matrix(mdsObj))
mdsObj$replicate <- rownames(mdsObj)
colnames(mdsObj) = c("dim1", "dim2", "replicate")
mdsObj = merge(mdsObj, sampleData, by.x = "replicate", by.y = "Replicate")

options(repr.plot.width = 5, repr.plot.height = 3)
ggscatter(mdsObj,
              x = "dim1",
              y = "dim2",
              color = "time",
              shape = "BF_status",
              size = 3.5,
              alpha = 0.8,
              ggtheme = theme_bw(),
              repel = "time",
         ) +
                theme(axis.text = element_text(size = 10), legend.title = elemen
t_blank(), axis.title = element_text(size = 12), legend.text = element_text(size
= 12)) +
                theme_bw() +
                labs ( x = "Dimension 1", y = "Dimension 2")

Combine annotation information with the dgeList object:
```

```
# Extract annotation for genes in the fit object
ann = subset(gene_ids, loc_ID %in% rownames(dgeList_fit))
# convert factors to characters
ann = data.frame(lapply(ann, as.character), stringsAsFactors=FALSE)
# align the fit object's rownames with gene ID's
ann = ann[match(rownames(dgeList_fit), ann$loc_ID),]
# convert factors to characters, again
ann <- data.frame(lapply(ann, as.character), stringsAsFactors=FALSE)
# Rename "FBgn_ID" as "GeneID"
colnames(ann) = c ("GeneID", "VB_ID", "GenBank_description")
# Check that the fit rownames match the annotation file's gene ID's
table(ann$GeneID==rownames(dgeList_fit))
# Add the annotations to the fit object in the "genes" slot
dgeList_fit$genes = ann
```

In [ ]:

```
##################################################################
###  Temporal transcriptional profile dynamics of the spermathecae
###    in response to mating and blood-feeding ##################
##################################################################

###Write the main contrasts###

BF.vs.virgin.time <- makeContrasts(Hr6 = BF_6Hr - Virgin,
                               Hr24 = BF_24Hr - Virgin,
                                 Hr72 = BF_72Hr - Virgin,
                                  levels = design_2)

NotBF.vs.virgin.time <- makeContrasts(Hr6 = NBF_6Hr - Virgin,
                               Hr24 = NBF_24Hr - Virgin,
                                 Hr72 = NBF_72Hr - Virgin,
                                  levels = design_2)
```

In [ ]:

```
##################################################################
###  Temporal transcriptional profile dynamics of the spermathecae
###    in response to mating and blood-feeding ###################
##################################################################

######The blood-fed vs. virgin comparison across time-points:

# 6 hour
Ftest.Hr6.BF.vs.virgin <- glmQLFTest(dgeList_fit, contrast = BF.vs.virgin.time[,
"Hr6"])
Ftest.Hr6.BF.vs.virgin.tTags <- topTags(Ftest.Hr6.BF.vs.virgin, n = NULL)
Ftest.Hr6.BF.vs.virgin.tTags.table <- Ftest.Hr6.BF.vs.virgin.tTags$table
Ftest.Hr6.BF.vs.virgin.tTags.table$sig = ifelse(Ftest.Hr6.BF.vs.virgin.tTags.tab
le$FDR < 0.05 & (Ftest.Hr6.BF.vs.virgin.tTags.table$logFC > 1 | Ftest.Hr6.BF.vs.
virgin.tTags.table$logFC < -1), "yes", "no")
Ftest.Hr6.BF.vs.virgin.tTags.table$time = "Hr6"
nrow(subset(Ftest.Hr6.BF.vs.virgin.tTags.table, sig == "yes"))

# 24 hour
Ftest.Hr24.BF.vs.virgin <- glmQLFTest(dgeList_fit, contrast = BF.vs.virgin.time
[,"Hr24"])
Ftest.Hr24.BF.vs.virgin.tTags <- topTags(Ftest.Hr24.BF.vs.virgin, n = NULL)
Ftest.Hr24.BF.vs.virgin.tTags.table <- Ftest.Hr24.BF.vs.virgin.tTags$table
Ftest.Hr24.BF.vs.virgin.tTags.table$sig = ifelse(Ftest.Hr24.BF.vs.virgin.tTags.t
able$FDR < 0.05 & (Ftest.Hr24.BF.vs.virgin.tTags.table$logFC > 1 | Ftest.Hr24.BF
.vs.virgin.tTags.table$logFC < -1), "yes", "no")
Ftest.Hr24.BF.vs.virgin.tTags.table$time = "Hr24"
nrow(subset(Ftest.Hr24.BF.vs.virgin.tTags.table, sig == "yes"))


# 72 hour
Ftest.Hr72.BF.vs.virgin <- glmQLFTest(dgeList_fit, contrast = BF.vs.virgin.time
[,"Hr72"])
Ftest.Hr72.BF.vs.virgin.tTags <- topTags(Ftest.Hr72.BF.vs.virgin, n = NULL)
Ftest.Hr72.BF.vs.virgin.tTags.table <- Ftest.Hr72.BF.vs.virgin.tTags$table
Ftest.Hr72.BF.vs.virgin.tTags.table$sig = ifelse(Ftest.Hr72.BF.vs.virgin.tTags.t
able$FDR < 0.05 & (Ftest.Hr72.BF.vs.virgin.tTags.table$logFC > 1 | Ftest.Hr72.BF
.vs.virgin.tTags.table$logFC < -1), "yes", "no")
Ftest.Hr72.BF.vs.virgin.tTags.table$time = "Hr72"
nrow(subset(Ftest.Hr72.BF.vs.virgin.tTags.table, sig == "yes"))

##Check the qq-plots:

options(repr.plot.width = 3, repr.plot.height = 2.2)
ggplot.qq(Ftest.Hr6.BF.vs.virgin.tTags.table$PValue, hit.idx = which(Ftest.Hr6.B
F.vs.virgin.tTags.table$FDR < 0.05)) + ggtitle("6Hr")
ggplot.qq(Ftest.Hr24.BF.vs.virgin.tTags.table$PValue, hit.idx = which(Ftest.Hr24
.BF.vs.virgin.tTags.table$FDR < 0.05)) + ggtitle("24Hr")
ggplot.qq(Ftest.Hr72.BF.vs.virgin.tTags.table$PValue, hit.idx = which(Ftest.Hr72
.BF.vs.virgin.tTags.table$FDR < 0.05)) + ggtitle("72Hr")
```

In [ ]:

```
#######################################################################
#######  Temporal transcriptional profile dynamics #############
### of the spermathecae in response to mating and blood-feeding ######
#######################################################################

##The not blood-fed vs. virgin comparison across time-points:
# 6 hour
Ftest.Hr6.NotBF.vs.virgin <- glmQLFTest(dgeList_fit, contrast = NotBF.vs.virgin.
time[,"Hr6"])
Ftest.Hr6.NotBF.vs.virgin.tTags <- topTags(Ftest.Hr6.NotBF.vs.virgin, n = NULL)
Ftest.Hr6.NotBF.vs.virgin.tTags.table <- Ftest.Hr6.NotBF.vs.virgin.tTags$table
Ftest.Hr6.NotBF.vs.virgin.tTags.table$sig = ifelse(Ftest.Hr6.NotBF.vs.virgin.tTa
gs.table$FDR < 0.05 & (Ftest.Hr6.NotBF.vs.virgin.tTags.table$logFC > 1 | Ftest.H
r6.NotBF.vs.virgin.tTags.table$logFC < -1), "yes", "no")
Ftest.Hr6.NotBF.vs.virgin.tTags.table$time = "Hr6"
nrow(subset(Ftest.Hr6.NotBF.vs.virgin.tTags.table, sig == "yes"))

# 24 hour
Ftest.Hr24.NotBF.vs.virgin <- glmQLFTest(dgeList_fit, contrast = NotBF.vs.virgin
.time[,"Hr24"])
Ftest.Hr24.NotBF.vs.virgin.tTags <- topTags(Ftest.Hr24.NotBF.vs.virgin, n = NULL
)
Ftest.Hr24.NotBF.vs.virgin.tTags.table <- Ftest.Hr24.NotBF.vs.virgin.tTags$table
Ftest.Hr24.NotBF.vs.virgin.tTags.table$sig = ifelse(Ftest.Hr24.NotBF.vs.virgin.t
Tags.table$FDR < 0.05 & (Ftest.Hr24.NotBF.vs.virgin.tTags.table$logFC > 1 | Ftes
t.Hr24.NotBF.vs.virgin.tTags.table$logFC < -1), "yes", "no")
Ftest.Hr24.NotBF.vs.virgin.tTags.table$time = "Hr24"
nrow(subset(Ftest.Hr24.NotBF.vs.virgin.tTags.table, sig == "yes"))

# 72 hour
Ftest.Hr72.NotBF.vs.virgin <- glmQLFTest(dgeList_fit, contrast = NotBF.vs.virgin
.time[,"Hr72"])
Ftest.Hr72.NotBF.vs.virgin.tTags <- topTags(Ftest.Hr72.NotBF.vs.virgin, n = NULL
)
Ftest.Hr72.NotBF.vs.virgin.tTags.table <- Ftest.Hr72.NotBF.vs.virgin.tTags$table
Ftest.Hr72.NotBF.vs.virgin.tTags.table$sig = ifelse(Ftest.Hr72.NotBF.vs.virgin.t
Tags.table$FDR < 0.05 & (Ftest.Hr72.NotBF.vs.virgin.tTags.table$logFC > 1 | Ftes
t.Hr72.NotBF.vs.virgin.tTags.table$logFC < -1), "yes", "no")
Ftest.Hr72.NotBF.vs.virgin.tTags.table$time = "Hr72"
nrow(subset(Ftest.Hr72.NotBF.vs.virgin.tTags.table, sig == "yes"))


##Check the qq-plots:

options(repr.plot.width = 3, repr.plot.height = 2.2)
ggplot.qq(Ftest.Hr6.NotBF.vs.virgin.tTags.table$PValue, hit.idx = which(Ftest.Hr
6.NotBF.vs.virgin.tTags.table$FDR < 0.05)) + ggtitle("6Hr")
ggplot.qq(Ftest.Hr24.NotBF.vs.virgin.tTags.table$PValue, hit.idx = which(Ftest.H
r24.NotBF.vs.virgin.tTags.table$FDR < 0.05)) + ggtitle("24Hr")
ggplot.qq(Ftest.Hr72.NotBF.vs.virgin.tTags.table$PValue, hit.idx = which(Ftest.H
r72.NotBF.vs.virgin.tTags.table$FDR < 0.05)) + ggtitle("72Hr")
```

In [ ]:

```
##Figure 3###

######################################   BF   #########################

####DE of BF per time point vs virgin####

Ftest.BF.vs.virgin.tTags.table = rbind(Ftest.Hr6.BF.vs.virgin.tTags.table, Ftest
.Hr24.BF.vs.virgin.tTags.table, Ftest.Hr72.BF.vs.virgin.tTags.table)
Ftest.BF.vs.virgin.tTags.table$comparison = "BF.vs.virgin"
Ftest.BF.vs.virgin.tTags.table$time = factor(Ftest.BF.vs.virgin.tTags.table$time
, levels = c("Hr6", "Hr24", "Hr72"))

options(repr.plot.width = 7, repr.plot.height = 3)
ggplot(Ftest.BF.vs.virgin.tTags.table, aes(logFC, -log10(PValue), colour = sig))
+
    geom_point(alpha = 0.7, size = 1) +
    facet_wrap(~time) +
#     theme_monokai_full() +
    scale_colour_manual(values = c("grey", "#d93b1b")) +
    geom_vline(xintercept = 0, linetype = "dashed", colour = "black") +
#     geom_text_repel(data = subset(Ftest.BF.vs.virgin.tTags.table, time == "Hr
6" & sig == "yes"), aes(logFC, -log10(PValue),label=SwissProt_BlastX_Descriptio
n),
#                     force = 50,
#                     inherit.aes = F,
#                     box.padding = unit(0.35, "lines"),
#                     point.padding = unit(0.5, "lines"),
#                     fontface = "bold",
#                     size = 3) +
    ggtitle("DE between blood-fed mated and \nvirgin females")


###Venn Diagrams BF vs virgin per time point####


## Upregaulted:

upregulated_candidates <- list(`6hr` = subset(Ftest.Hr6.BF.vs.virgin.tTags.table
, sig == "yes" & logFC > 1 & !is.na(GeneID))$GeneID,
                                `24hr` = subset(Ftest.Hr24.BF.vs.virgin.tTags.tab
le, sig == "yes" & logFC > 1 & !is.na(GeneID))$GeneID,
                                `72hr` = subset(Ftest.Hr72.BF.vs.virgin.tTags.tab
le, sig == "yes" & logFC > 1 & !is.na(GeneID))$GeneID)

upregulated_Vdiag<-venn.diagram(upregulated_candidates, NULL, fill=c("#a2a200",
"#ff6d81","#01906c"), alpha=c(0.75,0.75,0.75), cex = 1.5, cat.fontface= 6, cat.c
ex = 1, resolution = 100, main = "upregulated", main.cex = 1, cat.dist = c(0.05,
0.05, 0.05), main.dist = -0.9)

######
## Downregulated:

downregulated_candidates <- list(`6hr` = subset(Ftest.Hr6.BF.vs.virgin.tTags.tab
le, sig == "yes" & logFC < -1 & !is.na(GeneID))$GeneID,
                                `24hr` = subset(Ftest.Hr24.BF.vs.virgin.tTags.tab
le, sig == "yes" & logFC < -1 & !is.na(GeneID))$GeneID,
                                `72hr` = subset(Ftest.Hr72.BF.vs.virgin.tTags.tab
le, sig == "yes" & logFC < -1 & !is.na(GeneID))$GeneID)
```

```
downregulated_Vdiag<-venn.diagram(downregulated_candidates, NULL, fill=c("#a2a20
0","#ff6d81","#01906c"), alpha=c(0.75,0.75,0.75), cex = 1.5, cat.fontface= 6, ca
t.cex = 1, resolution = 100, main = "downregulated", main.cex = 1, cat.dist = c(
0.05, 0.05, 0.05), main.dist = -0.9)

options(repr.plot.width = 5, repr.plot.height = 2.75)
grid.arrange(gTree(children=upregulated_Vdiag), gTree(children=downregulated_Vdi
ag), ncol = 2, top = "DE genes between mated, blood-fed RT\n samples and virgin
 RT samples at three time-points")

#############################        NBF      ###########################

###Volcano Plot NBF vs virgin per time point

Ftest.NotBF.vs.virgin.tTags.table = rbind(Ftest.Hr6.NotBF.vs.virgin.tTags.table,
Ftest.Hr24.NotBF.vs.virgin.tTags.table, Ftest.Hr72.NotBF.vs.virgin.tTags.table)
Ftest.NotBF.vs.virgin.tTags.table$comparison = "NotBF.vs.virgin"
Ftest.NotBF.vs.virgin.tTags.table$time = factor(Ftest.NotBF.vs.virgin.tTags.tabl
e$time, levels = c("Hr6", "Hr24", "Hr72"))

options(repr.plot.width = 7, repr.plot.height = 3)
ggplot(Ftest.NotBF.vs.virgin.tTags.table, aes(logFC, -log10(PValue), colour = si
g)) +
    geom_point(alpha = 0.7, size = 1) +
    facet_wrap(~time) +
#     theme_monokai_full() +
    scale_colour_manual(values = c("grey", "#0075e1")) +
    geom_vline(xintercept = 0, linetype = "dashed", colour = "black") +
#     geom_text_repel(data = subset(Ftest.NotBF.vs.virgin.tTags.table, time ==
 "Hr6" & sig == "yes"), aes(logFC, -log10(PValue),label=SwissProt_BlastX_Descrip
tion),
#                     force = 50,
#                     inherit.aes = F,
#                     box.padding = unit(0.35, "lines"),
#                     point.padding = unit(0.5, "lines"),
#                     fontface = "bold",
#                     size = 3) +
    ggtitle("DE between nonblood-fed mated and \nvirgin females")

###
###Venn Diagrams NBF vs virgin per time point####

upregulated_candidates <- list(`6hr` = subset(Ftest.Hr6.NotBF.vs.virgin.tTags.ta
ble, sig == "yes" & logFC > 1 & !is.na(GeneID))$GeneID,
                               `24hr` = subset(Ftest.Hr24.NotBF.vs.virgin.tTags.
table, sig == "yes" & logFC > 1 & !is.na(GeneID))$GeneID,
                               `72hr` = subset(Ftest.Hr72.NotBF.vs.virgin.tTags.
table, sig == "yes" & logFC > 1 & !is.na(GeneID))$GeneID)

upregulated_Vdiag<-venn.diagram(upregulated_candidates, NULL, fill=c("#a2a200",
"#ff6d81","#01906c"), alpha=c(0.75,0.75,0.75), cex = 1.5, cat.fontface= 6, cat.c
ex = 1, resolution = 100, main = "upregulated", main.cex = 1, cat.dist = c(0.05,
0.05, 0.05), main.dist = -0.9)

######
## Downregulated:

downregulated_candidates <- list(`6hr` = subset(Ftest.Hr6.NotBF.vs.virgin.tTags.
table, sig == "yes" & logFC < -1 & !is.na(GeneID))$GeneID,
                               `24hr` = subset(Ftest.Hr24.NotBF.vs.virgin.tTags.
table, sig == "yes" & logFC < -1 & !is.na(GeneID))$GeneID,
```

```
                                      `72hr` = subset(Ftest.Hr72.NotBF.vs.virgin.tTags.
table, sig == "yes" & logFC < -1 & !is.na(GeneID))$GeneID)

downregulated_Vdiag<-venn.diagram(downregulated_candidates, NULL, fill=c("#a2a20
0","#ff6d81","#01906c"), alpha=c(0.75,0.75,0.75), cex = 1.5, cat.fontface= 6, ca
t.cex = 1, resolution = 100, main = "downregulated", main.cex = 1, cat.dist = c(
0.05, 0.05, 0.05), main.dist = -0.9)

options(repr.plot.width = 5, repr.plot.height =2.75)
grid.arrange(gTree(children=upregulated_Vdiag), gTree(children=downregulated_Vdi
ag), ncol = 2, top = "DE genes between mated, nonblood-fed RT\n samples and virg
in RT samples at three time-points")

###
###Bargraph
###FEED corresponds to the feeding status BF or NBF
Fig3E_stats<-lm(logFC~factor(FEED:t:REG), data=Bargraph)
summary(Fig3E_stats)
anova(Fig3E_stats)

require(multcomp)
pos.Fig3E_stats <- glht(Fig3E_stats, mcp("factor(FEED:t:REG)" = "Tukey"), decrea
sing = F)
summary(pos.Fig3E_stats)
```

In [ ]:

```
##########################################################
## Check male genes BF and NBF females in overall ##
##      and al time points data frames #######
######################################################

##Using a subset of genes of males mRNA from Degner's et al. paper 2019 male mRN
A potentially transfered
###during mating

#####Male genes in the combined dataset####
###Subset of male genes in BF table
mRNAmalesBF<-subset(Ftest.BF.vs.virgin.tTags.table, VB_ID %in% transferred.mRNAs
& sig == "yes")
mRNAmalesBF

write.table(mRNAmalesBF, file="mRNAmalesBF.csv", sep=",")

###Subset of male genes in NBF table
mRNAmalesNBF<-subset(Ftest.NotBF.vs.virgin.tTags.table, VB_ID %in% transferred.m
RNAs & sig == "yes")
mRNAmalesNBF

write.table(mRNAmalesNBF, file="mRNAmalesNBF.csv", sep=",")


########### Male genes at the different time points BF#####
mRNAmalesBF6<-subset(Ftest.Hr6.BF.vs.virgin.tTags.table, VB_ID %in% transferred.
mRNAs & sig == "yes")
mRNAmalesBF6

write.table(mRNAmalesBF6, file="mRNAmalesBF6.csv", sep=",")

mRNAmalesBF24<-subset(Ftest.Hr24.BF.vs.virgin.tTags.table, VB_ID %in% transferre
d.mRNAs & sig == "yes")
mRNAmalesBF24

write.table(mRNAmalesBF24, file="mRNAmalesBF24.csv", sep=",")

mRNAmalesBF72<-subset(Ftest.Hr72.BF.vs.virgin.tTags.table, VB_ID %in% transferre
d.mRNAs & sig == "yes")
mRNAmalesBF72

write.table(mRNAmalesBF72, file="mRNAmalesBF72.csv", sep=",")

########### Male genes at the different time points NBF#####

mRNAmalesNBF6<-subset(Ftest.Hr6.NotBF.vs.virgin.tTags.table, VB_ID %in% transfer
red.mRNAs & sig == "yes")
mRNAmalesNBF6

write.table(mRNAmalesNBF6, file="mRNAmalesNBF6.csv", sep=",")

mRNAmalesNBF24<-subset(Ftest.Hr24.NotBF.vs.virgin.tTags.table, VB_ID %in% transf
erred.mRNAs & sig == "yes")
mRNAmalesNBF24

write.table(mRNAmalesBF24, file="mRNAmalesNBF24.csv", sep=",")

mRNAmalesNBF72<-subset(Ftest.Hr72.NotBF.vs.virgin.tTags.table, VB_ID %in% transf
```

```
erred.mRNAs & sig == "yes")
mRNAmalesNBF72

write.table(mRNAmalesNBF72, file="mRNAmalesNBF72.csv", sep=",")
```

In [ ]:

```
###########################################################
####### Blood-feeding after mating induces ##
######  a transcriptional response in the spermathecae ##
#########################################################

BF.vs.NotBF.time <- makeContrasts(Hr6 = BF_6Hr - NBF_6Hr,
                               Hr24 = BF_24Hr - NBF_24Hr,
                                 Hr72 = BF_72Hr - NBF_72Hr,
                                  levels = design_2)

##The blood-fed vs. nonblood-fed comparison across time-points:

# 6 hour
Ftest.Hr6.BF.vs.NotBF <- glmQLFTest(dgeList_fit, contrast = BF.vs.NotBF.time[,"H
r6"])
Ftest.Hr6.BF.vs.NotBF.tTags <- topTags(Ftest.Hr6.BF.vs.NotBF, n = NULL)
Ftest.Hr6.BF.vs.NotBF.tTags.table <- Ftest.Hr6.BF.vs.NotBF.tTags$table
Ftest.Hr6.BF.vs.NotBF.tTags.table$sig = ifelse(Ftest.Hr6.BF.vs.NotBF.tTags.table
$FDR < 0.05 & (Ftest.Hr6.BF.vs.NotBF.tTags.table$logFC > 1 | Ftest.Hr6.BF.vs.Not
BF.tTags.table$logFC < -1), "yes", "no")
Ftest.Hr6.BF.vs.NotBF.tTags.table$time = "Hr6"
nrow(subset(Ftest.Hr6.BF.vs.NotBF.tTags.table, sig == "yes"))

# 24 hour
Ftest.Hr24.BF.vs.NotBF <- glmQLFTest(dgeList_fit, contrast = BF.vs.NotBF.time[,
"Hr24"])
Ftest.Hr24.BF.vs.NotBF.tTags <- topTags(Ftest.Hr24.BF.vs.NotBF, n = NULL)
Ftest.Hr24.BF.vs.NotBF.tTags.table <- Ftest.Hr24.BF.vs.NotBF.tTags$table
Ftest.Hr24.BF.vs.NotBF.tTags.table$sig = ifelse(Ftest.Hr24.BF.vs.NotBF.tTags.tab
le$FDR < 0.05 & (Ftest.Hr24.BF.vs.NotBF.tTags.table$logFC > 1 | Ftest.Hr24.BF.vs
.NotBF.tTags.table$logFC < -1), "yes", "no")
Ftest.Hr24.BF.vs.NotBF.tTags.table$time = "Hr24"
nrow(subset(Ftest.Hr24.BF.vs.NotBF.tTags.table, sig == "yes"))

# 72 hour
Ftest.Hr72.BF.vs.NotBF <- glmQLFTest(dgeList_fit, contrast = BF.vs.NotBF.time[,
"Hr72"])
Ftest.Hr72.BF.vs.NotBF.tTags <- topTags(Ftest.Hr72.BF.vs.NotBF, n = NULL)
Ftest.Hr72.BF.vs.NotBF.tTags.table <- Ftest.Hr72.BF.vs.NotBF.tTags$table
Ftest.Hr72.BF.vs.NotBF.tTags.table$sig = ifelse(Ftest.Hr72.BF.vs.NotBF.tTags.tab
le$FDR < 0.05 & (Ftest.Hr72.BF.vs.NotBF.tTags.table$logFC > 1 | Ftest.Hr72.BF.vs
.NotBF.tTags.table$logFC < -1), "yes", "no")
Ftest.Hr72.BF.vs.NotBF.tTags.table$time = "Hr72"
nrow(subset(Ftest.Hr72.BF.vs.NotBF.tTags.table, sig == "yes"))

##Check the qq-plots:

options(repr.plot.width = 3, repr.plot.height = 2.2)
ggplot.qq(Ftest.Hr6.BF.vs.NotBF.tTags.table$PValue, hit.idx = which(Ftest.Hr6.BF
.vs.NotBF.tTags.table$FDR < 0.05)) + ggtitle("6Hr")
ggplot.qq(Ftest.Hr24.BF.vs.NotBF.tTags.table$PValue, hit.idx = which(Ftest.Hr24.
BF.vs.NotBF.tTags.table$FDR < 0.05)) + ggtitle("24Hr")
ggplot.qq(Ftest.Hr72.BF.vs.NotBF.tTags.table$PValue, hit.idx = which(Ftest.Hr72.
BF.vs.NotBF.tTags.table$FDR < 0.05)) + ggtitle("72Hr")
```

In [ ]:

```
##############################################################
####### Blood-feeding after mating induces ##
######  a transcriptional response in the spermathecae ##
############################################################

##Figure 4##
## A ##
######Here is a function to make individual gene plots####

genePlot <- function(tpmTable, gene) {
  aael_id <- subset(locID_to_aaelID, loc_ID == gene)$active_id
  description <- subset(gene_ids, loc_ID == gene)$GenBank_description
  tmp <- subset(tpmTable, gene_id == gene)
  tmp.se <- summarySE(data = tmp, measurevar = "TPM", groupvars = c("gene_id",
"Sample", "mating_status", "BF_status", "time"))
  tmp.se$time = factor(tmp.se$time, levels = c("Virgin", "6Hr", "24Hr", "72Hr"))

  p <-ggplot() +
    geom_point(data = tmp.se, aes(time, TPM, colour = BF_status), position = pos
ition_dodge(.4)) +
    #     facet_grid(.~source) +
    geom_line(data = tmp.se, aes(time, TPM, group = BF_status, colour = BF_statu
s), position=position_dodge(.4)) +
    scale_colour_manual(values = c("#e52ba5","#a3c40b", "black")) +
    geom_errorbar(data = tmp.se, aes(time, colour = BF_status, ymin=TPM-se, ymax
=TPM+se), width=.2, position=position_dodge(.4), size = 0.5) +
    labs(title = paste(aael_id, " (", gene, ")", sep = ""), subtitle = paste(des
cription, sep = "\n")) +
    theme(axis.title.x = element_blank()) +
    theme_bw()

  return(p)
}

##B##
###Volcano Plot##

Ftest.BF.vs.NotBF.tTags.table = rbind(Ftest.Hr6.BF.vs.NotBF.tTags.table, Ftest.H
r24.BF.vs.NotBF.tTags.table, Ftest.Hr72.BF.vs.NotBF.tTags.table)
Ftest.BF.vs.NotBF.tTags.table$comparison = "BF.vs.NotBF"
Ftest.BF.vs.NotBF.tTags.table$time = factor(Ftest.BF.vs.NotBF.tTags.table$time,
levels = c("Hr6", "Hr24", "Hr72"))

options(repr.plot.width = 7, repr.plot.height = 3)
ggplot(Ftest.BF.vs.NotBF.tTags.table, aes(logFC, -log10(PValue), colour = sig))
+
    geom_point(alpha = 0.7, size = 1) +
    facet_wrap(~time) +
#     theme_monokai_full() +
    scale_colour_manual(values = c("grey", "#ffa16a")) +
    geom_vline(xintercept = 0, linetype = "dashed", colour = "black") +
#     geom_text_repel(data = subset(Ftest.BF.vs.NotBF.tTags.table, time == "Hr6"
& sig == "yes"), aes(logFC, -log10(PValue),label=SwissProt_BlastX_Description),
#                     force = 50,
#                     inherit.aes = F,
#                     box.padding = unit(0.35, "lines"),
#                     point.padding = unit(0.5, "lines"),
#                     fontface = "bold",
#                     size = 3) +
```

```
    ggtitle("DE between blod-fed and \nnonblood-fed mated females")
# ggsave("Results/Figures/BF.vs.NotBF_VolcanoPlots.pdf", width = 6, height = 3)
```

In [ ]:

```
###########################
##Gene Ontology analysis:
############################
##Using the GOseq package, the GO assignment for each gene and gene lengths are
 required. Load GO assignments from previous pipeline:

GO_info = read.csv("Trinotate_report.xls.gene_ontology", header=F, row.names=1,
sep = "\t", stringsAsFactors=F)
##Load the transcripts lengths, then convert to gene lengths:

# Load transcript lengths:
VB_trans_lengths = read.csv("AaegL5_VB.gtf_transcript_lengths.txt", header = F,
sep = " ")
# select transcrip IDs column and length column only:
tmp.df = subset(VB_trans_lengths, select = c("V1", "V2"))
tmp.df$V1 = gsub("-R.*", "", tmp.df$V1)
colnames(tmp.df) = c("gene_id", "length")
# For each gene ID entry, select the entry with largest value in the "length" co
lumn:
VB_gene_lengths = aggregate(length ~ gene_id, tmp.df, max)
# assign gene names to rownames
rownames(VB_gene_lengths) <- VB_gene_lengths$gene_id
# # select the length column only
VB_gene_lengths = subset(VB_gene_lengths, select = "length")
# remove un-needed intermediate objects
rm(tmp.df)

##Extract the relevant GO terms from the info fields:

GO_info_listed = apply(GO_info, 1, function(x) unlist(strsplit(x,',')))
names(GO_info_listed) = rownames(GO_info)
features_with_GO = rownames(GO_info)
lengths_features_with_GO = VB_gene_lengths[features_with_GO,]

##Here's the function to extract GO term descriptions:

get_GO_term_descr =  function(x) {
  d = 'none';
  go_info = GOTERM[[x]];
  if (length(go_info) >0) { d = paste(Ontology(go_info), Term(go_info), sep=' '
);}
  return(d);
}
```

In [ ]:

```
##GO analysis for each gene set###

############Overall NBF Upregulated Genes  ###############

NBF6hrUp<-rownames(subset(Ftest.Hr6.NotBF.vs.virgin.tTags.table, FDR<0.05  & log
FC>1 ))
NBF24hrUp<-rownames(subset(Ftest.Hr24.NotBF.vs.virgin.tTags.table, FDR<0.05  & l
ogFC>1 ))
NBF72hrUp<-rownames(subset(Ftest.Hr72.NotBF.vs.virgin.tTags.table, FDR<0.05  & l
ogFC>1 ))

NBF_ALLTIMESUP<-unique(c(NBF6hrUp,NBF24hrUp,NBF72hrUp))
NBF_ALLTIMESUP


geneSet <- subset(locID_to_aaelID, loc_ID %in% NBF_ALLTIMESUP)$active_id
options(repr.plot.width = 6, repr.plot.height = 3)
cat_genes_vec = as.integer(features_with_GO %in% geneSet)
pwf=nullp(cat_genes_vec,bias.data=lengths_features_with_GO)
rownames(pwf) = names(GO_info_listed)
options(repr.plot.width = 6, repr.plot.height = 3)
cat_genes_vec = as.integer(features_with_GO %in% geneSet)
pwf=nullp(cat_genes_vec,bias.data=lengths_features_with_GO)
rownames(pwf) = names(GO_info_listed)
cat_genes_vec = as.integer(features_with_GO %in% geneSet)
pwf$DEgenes = cat_genes_vec
res = goseq(pwf,gene2cat=GO_info_listed)
pvals = res$over_represented_pvalue
pvals[pvals > 1 -1e-10] = 1-1e-10
q = qvalue(pvals)
res$over_represented_FDR = q$qvalues
enrich_result_table = res[res$over_represented_pvalue<=0.05,]
descr = unlist(lapply(enrich_result_table$category, get_GO_term_descr))
enrich_result_table$go_term = descr

subset(enrich_result_table, over_represented_FDR < 0.05)

write.table(enrich_result_table, "GO_NBF_UP_GENRAL_ANALYSIS.csv",  sep = ",")
rm(enrich_result_table)



############Overall NBF Downregulated Genes###############

NBF6hrDown<-rownames(subset(Ftest.Hr6.NotBF.vs.virgin.tTags.table, FDR<0.05  & l
ogFC< 1 ))
NBF24hrDown<-rownames(subset(Ftest.Hr24.NotBF.vs.virgin.tTags.table, FDR<0.05  &
logFC< 1 ))
NBF72hrDown<-rownames(subset(Ftest.Hr72.NotBF.vs.virgin.tTags.table, FDR<0.05  &
logFC< 1 ))


NBF_ALLTIMESDOWN<-unique(c(NBF6hrDown,NBF24hrDown,NBF72hrDown))
NBF_ALLTIMESDOWN

geneSet <- subset(locID_to_aaelID, loc_ID %in% NBF_ALLTIMESDOWN)$active_id
options(repr.plot.width = 6, repr.plot.height = 3)
cat_genes_vec = as.integer(features_with_GO %in% geneSet)
pwf=nullp(cat_genes_vec,bias.data=lengths_features_with_GO)
```

```
rownames(pwf) = names(GO_info_listed)
options(repr.plot.width = 6, repr.plot.height = 3)
cat_genes_vec = as.integer(features_with_GO %in% geneSet)
pwf=nullp(cat_genes_vec,bias.data=lengths_features_with_GO)
rownames(pwf) = names(GO_info_listed)
cat_genes_vec = as.integer(features_with_GO %in% geneSet)
pwf$DEgenes = cat_genes_vec
res = goseq(pwf,gene2cat=GO_info_listed)
pvals = res$over_represented_pvalue
pvals[pvals > 1 -1e-10] = 1-1e-10
q = qvalue(pvals)
res$over_represented_FDR = q$qvalues
enrich_result_table = res[res$over_represented_pvalue<=0.05,]
descr = unlist(lapply(enrich_result_table$category, get_GO_term_descr))
enrich_result_table$go_term = descr

subset(enrich_result_table, over_represented_FDR < 0.05)

write.table(enrich_result_table, "GO_NBF_DOWN_GENRAL_ANALYSIS.csv",  sep = ",")
rm(enrich_result_table)


############Overall BF Upregulated Genes ###############

BF6hrUp<-rownames(subset(Ftest.Hr6.BF.vs.virgin.tTags.table, FDR<0.05  & logFC>1
))
BF24hrUp<-rownames(subset(Ftest.Hr24.BF.vs.virgin.tTags.table, FDR<0.05  & logFC
>1 ))
BF72hrUp<-rownames(subset(Ftest.Hr72.BF.vs.virgin.tTags.table, FDR<0.05  & logFC
>1 ))

BF_ALLTIMESUP<-unique(c(BF6hrUp,BF24hrUp,BF72hrUp))
BF_ALLTIMESUP


geneSet <- subset(locID_to_aaelID, loc_ID %in% BF_ALLTIMESUP)$active_id
options(repr.plot.width = 6, repr.plot.height = 3)
cat_genes_vec = as.integer(features_with_GO %in% geneSet)
pwf=nullp(cat_genes_vec,bias.data=lengths_features_with_GO)
rownames(pwf) = names(GO_info_listed)
options(repr.plot.width = 6, repr.plot.height = 3)
cat_genes_vec = as.integer(features_with_GO %in% geneSet)
pwf=nullp(cat_genes_vec,bias.data=lengths_features_with_GO)
rownames(pwf) = names(GO_info_listed)
cat_genes_vec = as.integer(features_with_GO %in% geneSet)
pwf$DEgenes = cat_genes_vec
res = goseq(pwf,gene2cat=GO_info_listed)
pvals = res$over_represented_pvalue
pvals[pvals > 1 -1e-10] = 1-1e-10
q = qvalue(pvals)
res$over_represented_FDR = q$qvalues
enrich_result_table = res[res$over_represented_pvalue<=0.05,]
descr = unlist(lapply(enrich_result_table$category, get_GO_term_descr))
enrich_result_table$go_term = descr

subset(enrich_result_table, over_represented_FDR < 0.05)

write.table(enrich_result_table, "GO_BF_UP_GENRAL_ANALYSIS.csv",  sep = ",")
rm(enrich_result_table)

############Overall BF Downregulated Genes###############
```

```
##Subset of upregulated genes at all time points in BF females###
BF6hrDown<-rownames(subset(Ftest.Hr6.BF.vs.virgin.tTags.table, FDR<0.05  & logFC
< 1 ))
BF24hrDown<-rownames(subset(Ftest.Hr24.BF.vs.virgin.tTags.table, FDR<0.05  & log
FC< 1 ))
BF72hrDown<-rownames(subset(Ftest.Hr72.BF.vs.virgin.tTags.table, FDR<0.05  & log
FC< 1 ))

###Combine upregulated genes at all time points in BF females###
BF_ALLTIMESDOWN<-unique(c(BF6hrDown,BF24hrDown,BF72hrDown))
BF_ALLTIMESDOWN

geneSet <- subset(locID_to_aaelID, loc_ID %in% BF_ALLTIMESDOWN)$active_id
options(repr.plot.width = 6, repr.plot.height = 3)
cat_genes_vec = as.integer(features_with_GO %in% geneSet)
pwf=nullp(cat_genes_vec,bias.data=lengths_features_with_GO)
rownames(pwf) = names(GO_info_listed)
options(repr.plot.width = 6, repr.plot.height = 3)
cat_genes_vec = as.integer(features_with_GO %in% geneSet)
pwf=nullp(cat_genes_vec,bias.data=lengths_features_with_GO)
rownames(pwf) = names(GO_info_listed)
cat_genes_vec = as.integer(features_with_GO %in% geneSet)
pwf$DEgenes = cat_genes_vec
res = goseq(pwf,gene2cat=GO_info_listed)
pvals = res$over_represented_pvalue
pvals[pvals > 1 -1e-10] = 1-1e-10
q = qvalue(pvals)
res$over_represented_FDR = q$qvalues
enrich_result_table = res[res$over_represented_pvalue<=0.05,]
descr = unlist(lapply(enrich_result_table$category, get_GO_term_descr))
enrich_result_table$go_term = descr

subset(enrich_result_table, over_represented_FDR < 0.05)
write.table(enrich_result_table, "GO_BF_DOWN_GENRAL_ANALYSIS.csv",  sep = ",")

rm(enrich_result_table)
```

In [ ]:

```
#######GENE ONTOLOGY ANALYSIS PER TIME POINT##################


################### GO NBF TIME POINTS ##########

##Upregulated NBF
###6 hrs Upregulated NBF

NBFUp_6hrs <- rownames(subset(Ftest.Hr6.NotBF.vs.virgin.tTags.table, FDR<0.05  &
logFC > 1))

head(NBFUp_6hrs)
geneSet <- subset(locID_to_aaelID, loc_ID %in% NBFUp_6hrs)$active_id
options(repr.plot.width = 6, repr.plot.height = 3)
cat_genes_vec = as.integer(features_with_GO %in% geneSet)
pwf=nullp(cat_genes_vec,bias.data=lengths_features_with_GO)
rownames(pwf) = names(GO_info_listed)
cat_genes_vec = as.integer(features_with_GO %in% geneSet)
pwf$DEgenes = cat_genes_vec
res = goseq(pwf,gene2cat=GO_info_listed)
pvals = res$over_represented_pvalue
pvals[pvals > 1 -1e-10] = 1-1e-10
q = qvalue(pvals)
res$over_represented_FDR = q$qvalues
enrich_result_table = res[res$over_represented_pvalue<=0.05,]
descr = unlist(lapply(enrich_result_table$category, get_GO_term_descr))
enrich_result_table$go_term = descr
head(enrich_result_table)

###24 hrs Upregulated NBF

NBFUp_24hrs<- rownames(subset(Ftest.Hr24.NotBF.vs.virgin.tTags.table, FDR<0.05
& logFC > 1 ))

geneSet <- subset(locID_to_aaelID, loc_ID %in% NBFUp_24hrs)$active_id
options(repr.plot.width = 6, repr.plot.height = 3)
cat_genes_vec = as.integer(features_with_GO %in% geneSet)
pwf=nullp(cat_genes_vec,bias.data=lengths_features_with_GO)
rownames(pwf) = names(GO_info_listed)
cat_genes_vec = as.integer(features_with_GO %in% geneSet)
pwf$DEgenes = cat_genes_vec
res = goseq(pwf,gene2cat=GO_info_listed)
pvals = res$over_represented_pvalue
pvals[pvals > 1 -1e-10] = 1-1e-10
q = qvalue(pvals)
res$over_represented_FDR = q$qvalues
enrich_result_table = res[res$over_represented_pvalue<=0.05,]
descr = unlist(lapply(enrich_result_table$category, get_GO_term_descr))
enrich_result_table$go_term = descr
head(enrich_result_table)

###72 hrs Upregulated NBF

NBFUp_72hrs <- rownames(subset(Ftest.Hr72.NotBF.vs.virgin.tTags.table, FDR<0.05
& logFC > 1 ))

head(NBFUp_72hrs)
geneSet <- subset(locID_to_aaelID, loc_ID %in% NBFUp_72hrs)$active_id
options(repr.plot.width = 6, repr.plot.height = 3)
```

```
cat_genes_vec = as.integer(features_with_GO %in% geneSet)
pwf=nullp(cat_genes_vec,bias.data=lengths_features_with_GO)
rownames(pwf) = names(GO_info_listed)
cat_genes_vec = as.integer(features_with_GO %in% geneSet)
pwf$DEgenes = cat_genes_vec
res = goseq(pwf,gene2cat=GO_info_listed)
pvals = res$over_represented_pvalue
pvals[pvals > 1 -1e-10] = 1-1e-10
q = qvalue(pvals)
res$over_represented_FDR = q$qvalues
enrich_result_table = res[res$over_represented_pvalue<=0.05,]
descr = unlist(lapply(enrich_result_table$category, get_GO_term_descr))
enrich_result_table$go_term = descr
head(enrich_result_table)

##Downregulated NBF

###6 Hrs Downregulated NBF

NBFDown_6hrs <- rownames(subset(Ftest.Hr6.NotBF.vs.virgin.tTags.table, FDR<0.05
& logFC < 1 ))

head(NBFDown_6hrs)
geneSet <- subset(locID_to_aaelID, loc_ID %in% NBFDown_6hrs)$active_id
options(repr.plot.width = 6, repr.plot.height = 3)
cat_genes_vec = as.integer(features_with_GO %in% geneSet)
pwf=nullp(cat_genes_vec,bias.data=lengths_features_with_GO)
rownames(pwf) = names(GO_info_listed)
cat_genes_vec = as.integer(features_with_GO %in% geneSet)
pwf$DEgenes = cat_genes_vec
res = goseq(pwf,gene2cat=GO_info_listed)
pvals = res$over_represented_pvalue
pvals[pvals > 1 -1e-10] = 1-1e-10
q = qvalue(pvals)
res$over_represented_FDR = q$qvalues
enrich_result_table = res[res$over_represented_pvalue<=0.05,]
descr = unlist(lapply(enrich_result_table$category, get_GO_term_descr))
enrich_result_table$go_term = descr
head(enrich_result_table)


###24 Hrs Downregulated NBF####

NBFDown_24hrs <- rownames(subset(Ftest.Hr24.NotBF.vs.virgin.tTags.table, FDR<0.0
5 & logFC < 1 ))

head(NBFDown_24hrs)
geneSet <- subset(locID_to_aaelID, loc_ID %in% NBFDown_24hrs)$active_id
options(repr.plot.width = 6, repr.plot.height = 3)
cat_genes_vec = as.integer(features_with_GO %in% geneSet)
pwf=nullp(cat_genes_vec,bias.data=lengths_features_with_GO)
rownames(pwf) = names(GO_info_listed)
cat_genes_vec = as.integer(features_with_GO %in% geneSet)
pwf$DEgenes = cat_genes_vec
res = goseq(pwf,gene2cat=GO_info_listed)
pvals = res$over_represented_pvalue
pvals[pvals > 1 -1e-10] = 1-1e-10
q = qvalue(pvals)
res$over_represented_FDR = q$qvalues
enrich_result_table = res[res$over_represented_pvalue<=0.05,]
descr = unlist(lapply(enrich_result_table$category, get_GO_term_descr))
```

```
enrich_result_table$go_term = descr
head(enrich_result_table)


### 72 hrs Downregulated NBF

NBFDown_72hrs <- rownames(subset(Ftest.Hr72.NotBF.vs.virgin.tTags.table, FDR<0.0
5 & logFC < 1 ))

head(NBFDown_72hrs)
geneSet <- subset(locID_to_aaelID, loc_ID %in% NBFDown_72hrs)$active_id
options(repr.plot.width = 6, repr.plot.height = 3)
cat_genes_vec = as.integer(features_with_GO %in% geneSet)
pwf=nullp(cat_genes_vec,bias.data=lengths_features_with_GO)
rownames(pwf) = names(GO_info_listed)
cat_genes_vec = as.integer(features_with_GO %in% geneSet)
pwf$DEgenes = cat_genes_vec
res = goseq(pwf,gene2cat=GO_info_listed)
pvals = res$over_represented_pvalue
pvals[pvals > 1 -1e-10] = 1-1e-10
q = qvalue(pvals)
res$over_represented_FDR = q$qvalues
enrich_result_table = res[res$over_represented_pvalue<=0.05,]
descr = unlist(lapply(enrich_result_table$category, get_GO_term_descr))
enrich_result_table$go_term = descr
head(enrich_result_table)
```

In [ ]:

```
################### GO BF TIME POINTS ##########

##Upregulated BF
###6 hrs Upregulated BF

BFUp_6hrs <- rownames(subset(Ftest.Hr6.BF.vs.virgin.tTags.table, FDR<0.05  & log
FC > 1 ))

head(BFUp_6hrs)
geneSet <- subset(locID_to_aaelID, loc_ID %in% BFUp_6hrs)$active_id
options(repr.plot.width = 6, repr.plot.height = 3)
cat_genes_vec = as.integer(features_with_GO %in% geneSet)
pwf=nullp(cat_genes_vec,bias.data=lengths_features_with_GO)
rownames(pwf) = names(GO_info_listed)
cat_genes_vec = as.integer(features_with_GO %in% geneSet)
pwf$DEgenes = cat_genes_vec
res = goseq(pwf,gene2cat=GO_info_listed)
pvals = res$over_represented_pvalue
pvals[pvals > 1 -1e-10] = 1-1e-10
q = qvalue(pvals)
res$over_represented_FDR = q$qvalues
enrich_result_table = res[res$over_represented_pvalue<=0.05,]
descr = unlist(lapply(enrich_result_table$category, get_GO_term_descr))
enrich_result_table$go_term = descr
head(enrich_result_table)


### 24 hrs BF Upregulated

BFUp_24hrs <- rownames(subset(Ftest.Hr24.BF.vs.virgin.tTags.table, FDR<0.05  & l
ogFC > 1 ))

head(BFUp_24hrs)
geneSet <- subset(locID_to_aaelID, loc_ID %in% BFUp_24hrs)$active_id
options(repr.plot.width = 6, repr.plot.height = 3)
cat_genes_vec = as.integer(features_with_GO %in% geneSet)
pwf=nullp(cat_genes_vec,bias.data=lengths_features_with_GO)
rownames(pwf) = names(GO_info_listed)
cat_genes_vec = as.integer(features_with_GO %in% geneSet)
pwf$DEgenes = cat_genes_vec
res = goseq(pwf,gene2cat=GO_info_listed)
pvals = res$over_represented_pvalue
pvals[pvals > 1 -1e-10] = 1-1e-10
q = qvalue(pvals)
res$over_represented_FDR = q$qvalues
enrich_result_table = res[res$over_represented_pvalue<=0.05,]
descr = unlist(lapply(enrich_result_table$category, get_GO_term_descr))
enrich_result_table$go_term = descr
head(enrich_result_table)


###72 hrs Upregulated BF

BFUp_72hrs<- rownames(subset(Ftest.Hr72.BF.vs.virgin.tTags.table, FDR<0.05 & log
FC > 1 ))

head(BFUp_72hrs)
geneSet <- subset(locID_to_aaelID, loc_ID %in% BFUp_72hrs)$active_id
options(repr.plot.width = 6, repr.plot.height = 3)
```

```
cat_genes_vec = as.integer(features_with_GO %in% geneSet)
pwf=nullp(cat_genes_vec,bias.data=lengths_features_with_GO)
rownames(pwf) = names(GO_info_listed)
cat_genes_vec = as.integer(features_with_GO %in% geneSet)
pwf$DEgenes = cat_genes_vec
res = goseq(pwf,gene2cat=GO_info_listed)
pvals = res$over_represented_pvalue
pvals[pvals > 1 -1e-10] = 1-1e-10
q = qvalue(pvals)
res$over_represented_FDR = q$qvalues
enrich_result_table = res[res$over_represented_pvalue<=0.05,]
descr = unlist(lapply(enrich_result_table$category, get_GO_term_descr))
enrich_result_table$go_term = descr
head(enrich_result_table)


##Downregulated BF

###6 hrs Downregulated BF

BFDown_6hrs <- rownames(subset(Ftest.Hr6.BF.vs.virgin.tTags.table, FDR<0.05 & lo
gFC < 1 ))

geneSet <- subset(locID_to_aaelID, loc_ID %in% BFDown_6hrs )$active_id
options(repr.plot.width = 6, repr.plot.height = 3)
cat_genes_vec = as.integer(features_with_GO %in% geneSet)
pwf=nullp(cat_genes_vec,bias.data=lengths_features_with_GO)
rownames(pwf) = names(GO_info_listed)
cat_genes_vec = as.integer(features_with_GO %in% geneSet)
pwf$DEgenes = cat_genes_vec
res = goseq(pwf,gene2cat=GO_info_listed)
pvals = res$over_represented_pvalue
pvals[pvals > 1 -1e-10] = 1-1e-10
q = qvalue(pvals)
res$over_represented_FDR = q$qvalues
enrich_result_table = res[res$over_represented_pvalue<=0.05,]
descr = unlist(lapply(enrich_result_table$category, get_GO_term_descr))
enrich_result_table$go_term = descr
head(enrich_result_table)



###24 hrs Downregulated BF

BFDown_24hrs<- rownames(subset(Ftest.Hr24.BF.vs.virgin.tTags.table, FDR<0.05 & l
ogFC < 1 ))

head(BFDown_24hrs)
geneSet <- subset(locID_to_aaelID, loc_ID %in% BFDown_24hrs)$active_id
options(repr.plot.width = 6, repr.plot.height = 3)
cat_genes_vec = as.integer(features_with_GO %in% geneSet)
pwf=nullp(cat_genes_vec,bias.data=lengths_features_with_GO)
rownames(pwf) = names(GO_info_listed)
cat_genes_vec = as.integer(features_with_GO %in% geneSet)
pwf$DEgenes = cat_genes_vec
res = goseq(pwf,gene2cat=GO_info_listed)
pvals = res$over_represented_pvalue
pvals[pvals > 1 -1e-10] = 1-1e-10
q = qvalue(pvals)
res$over_represented_FDR = q$qvalues
enrich_result_table = res[res$over_represented_pvalue<=0.05,]
```

```
descr = unlist(lapply(enrich_result_table$category, get_GO_term_descr))
enrich_result_table$go_term = descr
head(enrich_result_table)


###72 hrs Downregulated BF


BFDown_72hrs <- rownames(subset(Ftest.Hr72.BF.vs.virgin.tTags.table, FDR<0.05 &
logFC < 1 ))

head(BFDown_72hrs)
geneSet <- subset(locID_to_aaelID, loc_ID %in% BFDown_72hrs)$active_id
options(repr.plot.width = 6, repr.plot.height = 3)
cat_genes_vec = as.integer(features_with_GO %in% geneSet)
pwf=nullp(cat_genes_vec,bias.data=lengths_features_with_GO)
rownames(pwf) = names(GO_info_listed)
cat_genes_vec = as.integer(features_with_GO %in% geneSet)
pwf$DEgenes = cat_genes_vec
res = goseq(pwf,gene2cat=GO_info_listed)
pvals = res$over_represented_pvalue
pvals[pvals > 1 -1e-10] = 1-1e-10
q = qvalue(pvals)
res$over_represented_FDR = q$qvalues
enrich_result_table = res[res$over_represented_pvalue<=0.05,]
descr = unlist(lapply(enrich_result_table$category, get_GO_term_descr))
enrich_result_table$go_term = descr

head(enrich_result_table)

write.table(enrich_result_table, "GO_BFvsVIRGIN72hoursDOWN.csv",  sep = ",")
```

In [ ]:

```
##########################################################
####Comparative expression patterns between the spermathecae
## and lowerreproductive tract tissues.####################
##########################################################

####Comparison with Alfonso-Parra et al (2016):


###Reanalyze the 2016 data to extract the logFC:

cpm_Alfonso <- cpm(counts_Alfonso)
thresh_Alfonso <- cpm_Alfonso > 5
keep_Alfonso <- rowSums(thresh_Alfonso) >= 2
countsKeep_Alfonso <- counts_Alfonso[keep_Alfonso,]

# countsKeep_qM <- subset(countsKeep_qM, rownames(countsKeep_qM) %!in% wierd_gen
es)
table(keep_Alfonso)
keep_Alfonso

dev.off()

##Make samples table:

replicates_column = colnames(counts_Alfonso)
samples_column = gsub("_\\d$", "", replicates_column)
sample.info.a = data.frame(sample = samples_column, replicate = replicates_colum
n)
sample.info.a$status = ifelse(sample.info.a$sample == "virgin", "virgin", "mate
d")

##Specify sampe grouping and design matrix

groups_Alfonso = factor(sample.info.a$sample)
design_Alfonso = model.matrix(~0+groups_Alfonso)
colnames(design_Alfonso) <- levels(groups_Alfonso)
rownames(design_Alfonso) <- sample.info.a$replicate

##Fit the model:

dgeList_Alfonso <- DGEList(counts = countsKeep_Alfonso, group = groups_Alfonso)
dgeList_Alfonso <- calcNormFactors(dgeList_Alfonso)
dgeList_Alfonso <- estimateDisp(dgeList_Alfonso, design_Alfonso)
# dgeList <- estimateGLMTagwiseDisp(dgeList, design)
dgeList_Alfonso_fit <- glmQLFit(dgeList_Alfonso, design_Alfonso)
summary(dgeList_Alfonso$tagwise.dispersion)

###Check some of the model parameters;

options(repr.plot.width = 9, repr.plot.height = 6)
par(mfrow=c(2,2))
# Biological coefficient of variation
plotBCV(dgeList_Alfonso)
# mean-variance trend
voom = voom(dgeList_Alfonso, design_Alfonso, plot=TRUE)
# QQ-plot
g.v <- gof(dgeList_Alfonso_fit, plot = T, pcutoff = 0.05, adjust = "holm")
# z.v <- zscoreGamma(g.v$gof.statistics,shape=g.v$df/2,scale=2)
# qqnorm(z.v); qqline(z.v, col = 4,lwd=1,lty=1)
```

```
# log2 transformed and normalize boxplot of counts across samples
boxplot(voom$E, xlab="", ylab="Log2 counts per million",las=2,main="Voom transfo
rmed logCPM")
abline(h=median(voom$E),col="blue")

rm(voom)

##Sample Grouping:

## Plot sample correlation
data = log2(countsKeep_Alfonso+1)
# colnames(data) = gsub("Female_", "", colnames(data))
data = as.matrix(data)
sample_cor = cor(data, method='pearson', use='pairwise.complete.obs')

options(repr.plot.width = 4, repr.plot.height = 3.5)
pheatmap(
  mat               = sample_cor,
  color             = inferno(50),
  border_color      = NA,
  show_colnames     = TRUE,
  show_rownames     = TRUE,
  #   filename          = "Figures/sample_correlations_all.pdf",
  #   width             = 6,
  #   height            = 5,
  fontsize          = 12
)
rm(data)
rm(sample_cor)

##Add annotation info to dgeList oject:

# Extract annotation for genes in the fit object
ann = subset(Annots, gene_id %in% rownames(dgeList_Alfonso_fit))
# convert factors to characters
ann = data.frame(lapply(ann, as.character), stringsAsFactors=FALSE)
# align the fit object's rownames with gene ID's
ann = ann[match(rownames(dgeList_Alfonso_fit), ann$gene_id),]
# convert factors to characters, again
ann <- data.frame(lapply(ann, as.character), stringsAsFactors=FALSE)
# Rename "FBgn_ID" as "GeneID"
colnames(ann) = c ("GeneID", "gene_name", "VectorBae_description", "SwissProt_Bl
astX_Description")
# Check that the fit rownames match the annotation file's gene ID's
table(ann$GeneID==rownames(dgeList_Alfonso_fit))
# Add the annotations to the fit object in the "genes" slot
dgeList_Alfonso_fit$genes = ann
rm(ann)

##Now define the relevant contrasts and perform DE tests::

hpm0.v.virgin <- makeContrasts(X0hpm-virgin,
                               levels=design_Alfonso)

Ftest.hpm0.v.virgin <- glmQLFTest(dgeList_Alfonso_fit, contrast = hpm0.v.virgin)
Ftest.hpm0.v.virgin.tTags <- topTags(Ftest.hpm0.v.virgin, n = NULL)
Ftest.hpm0.v.virgin.tTags.table <- Ftest.hpm0.v.virgin.tTags$table
Ftest.hpm0.v.virgin.tTags.table$sig = ifelse(Ftest.hpm0.v.virgin.tTags.table$FDR
< 0.05 & (Ftest.hpm0.v.virgin.tTags.table$logFC > 1 | Ftest.hpm0.v.virgin.tTags.
table$logFC < -1), "yes", "no")
Ftest.hpm0.v.virgin.tTags.table$time = "X0hpm"
```

```
Alfonso_0hpm_v_virgin_sigGene <- rownames(subset(Ftest.hpm0.v.virgin.tTags.table
, sig == "yes"))
nrow(subset(Ftest.hpm0.v.virgin.tTags.table, sig == "yes"))


hpm6.v.virgin <- makeContrasts(X6hpm-virgin,
                               levels=design_Alfonso)

Ftest.hpm6.v.virgin <- glmQLFTest(dgeList_Alfonso_fit, contrast = hpm6.v.virgin)
Ftest.hpm6.v.virgin.tTags <- topTags(Ftest.hpm6.v.virgin, n = NULL)
Ftest.hpm6.v.virgin.tTags.table <- Ftest.hpm6.v.virgin.tTags$table
Ftest.hpm6.v.virgin.tTags.table$sig = ifelse(Ftest.hpm6.v.virgin.tTags.table$FDR
< 0.01 & (Ftest.hpm6.v.virgin.tTags.table$logFC > 1 | Ftest.hpm6.v.virgin.tTags.
table$logFC < -1), "yes", "no")
Ftest.hpm6.v.virgin.tTags.table$time = "6hpm"
Alfonso_6hpm_v_virgin_sigGene <- rownames(subset(Ftest.hpm6.v.virgin.tTags.table
, sig == "yes"))
nrow(subset(Ftest.hpm6.v.virgin.tTags.table, sig == "yes"))

hpm24.v.virgin <- makeContrasts(X24hpm-virgin,
                                levels=design_Alfonso)

Ftest.hpm24.v.virgin <- glmQLFTest(dgeList_Alfonso_fit, contrast = hpm24.v.virgi
n)
Ftest.hpm24.v.virgin.tTags <- topTags(Ftest.hpm24.v.virgin, n = NULL)
Ftest.hpm24.v.virgin.tTags.table <- Ftest.hpm24.v.virgin.tTags$table
Ftest.hpm24.v.virgin.tTags.table$sig = ifelse(Ftest.hpm24.v.virgin.tTags.table$F
DR < 0.01 & (Ftest.hpm24.v.virgin.tTags.table$logFC > 1 | Ftest.hpm24.v.virgin.t
Tags.table$logFC < -1), "yes", "no")
Ftest.hpm24.v.virgin.tTags.table$time = "24hpm"
Alfonso_24hpm_v_virgin_sigGene <- rownames(subset(Ftest.hpm24.v.virgin.tTags.tab
le, sig == "yes"))
nrow(subset(Ftest.hpm24.v.virgin.tTags.table, sig == "yes"))

Alf.comb = rbind(Ftest.hpm0.v.virgin.tTags.table, Ftest.hpm6.v.virgin.tTags.tabl
e, Ftest.hpm24.v.virgin.tTags.table)
Alf.comb$time = factor(Alf.comb$time, levels = c("0hpm", "6hpm", "24hpm"))

options(repr.plot.width = 7, repr.plot.height = 3)
ggplot(Alf.comb, aes(logFC, -log10(PValue), colour = sig)) +
  geom_point(alpha = 0.7, size = 1) +
  facet_wrap(~time) +
  #     theme_monokai_full() +
  scale_colour_manual(values = c("grey", "#8277cc")) +
  geom_vline(xintercept = 0, linetype = "dashed") +
  ggtitle("Alfonso-Para et al. (2016) results")


##To compare the logFC from the two studies, we need to extrac the relevant part
s of the De tables:

### select relevant 6hpm data for blood fed:
tmp.Camargo_BF.6hpm = select(Ftest.Hr6.BF.vs.virgin.tTags.table, GeneID, VB_ID,
CC_logFC = logFC, CC_sig = sig, GenBank_description)
tmp.Camargo_BF.6hpm$data = "Blood-fed"

### select relevant 6hpm data for nonblood fed:
tmp.Camargo_NBF.6hpm = select(Ftest.Hr6.NotBF.vs.virgin.tTags.table, GeneID, VB_
ID, CC_logFC = logFC, CC_sig = sig, GenBank_description)
tmp.Camargo_NBF.6hpm$data = "Not blood-fed"
```

```
## combine 6hpm data from BF and NBF:
tmp.Camargo.6hpm = rbind(tmp.Camargo_BF.6hpm, tmp.Camargo_NBF.6hpm)

### select relevant 6hpm data from 2016 paper:
tmp.Alfonso.6hpm = select(Ftest.hpm6.v.virgin.tTags.table, GeneID, Al_logFC = lo
gFC, Al_sig = sig, time, VectorBae_description, SwissProt_BlastX_Description)

## merge current 6hpm study's data with 2016 6hpm data:
tmp6hr = merge(tmp.Camargo.6hpm, tmp.Alfonso.6hpm, by.x = "VB_ID", by.y = "GeneI
D", all = T)


### select relevant 24hpm data for blood fed:
tmp.Camargo_BF.24hpm = select(Ftest.Hr24.BF.vs.virgin.tTags.table, GeneID, VB_ID
, CC_logFC = logFC, CC_sig = sig, GenBank_description)
tmp.Camargo_BF.24hpm$data = "Blood-fed"

### select relevant 24hpm data for nonblood fed:
tmp.Camargo_NBF.24hpm = select(Ftest.Hr24.NotBF.vs.virgin.tTags.table, GeneID, V
B_ID, CC_logFC = logFC, CC_sig = sig, GenBank_description)
tmp.Camargo_NBF.24hpm$data = "Not blood-fed"

## combine 24hpm data from BF and NBF:
tmp.Camargo.24hpm = rbind(tmp.Camargo_BF.24hpm, tmp.Camargo_NBF.24hpm)

### select relevant 24hpm data from 20124 paper:
tmp.Alfonso.24hpm = select(Ftest.hpm24.v.virgin.tTags.table, GeneID, Al_logFC =
logFC, Al_sig = sig, time, VectorBae_description, SwissProt_BlastX_Description)

## merge current 24hpm study's data with 20124 24hpm data:
tmp24hr = merge(tmp.Camargo.24hpm, tmp.Alfonso.24hpm, by.x = "VB_ID", by.y = "Ge
neID", all = T)

combined_data = rbind(tmp6hr, tmp24hr)
combined_data$time = factor(combined_data$time, levels = c("6hpm", "24hpm"))

head(combined_data)
```

In [ ]:

```
################################################################################
####Comparative expression patterns between the spermathecae and lower
##reproductive tract tissues.##################################################
################################################################################

########Figure 5#####
##Plot the logFC coparison for both studies:

options(repr.plot.width = 7, repr.plot.height =6)
ggplot() +
  geom_point(data = subset(combined_data, CC_sig == "no" & Al_sig == "no"), aes(
CC_logFC, Al_logFC), colour = "gray", alpha = 0.5) +
  geom_point(data = subset(combined_data, CC_sig == "yes" & Al_sig == "yes"), ae
s(CC_logFC, Al_logFC), colour = "#464a99", alpha = 0.5) +
  geom_vline(xintercept = 0, linetype = "dashed") +
  geom_hline(yintercept = 0, linetype = "dashed") +
  geom_abline(intercept = 0, slope = 1, linetype = "dashed", colour = "gray", al
pha = 0.5) +
  facet_grid(time~data) +
  geom_text_repel(data = subset(combined_data, Al_logFC > 5 & CC_sig == "yes" &
Al_sig == "yes"), aes(CC_logFC, Al_logFC,label=VectorBae_description),
                  force = 50,
                  inherit.aes = F,
                  box.padding = unit(0.35, "lines"),
                  point.padding = unit(0.5, "lines"),
                  fontface = "bold",
                  size = 3) +
  labs(x = "log fold-change (current study)", y = "log fold-change (Alfonso-Para
et al 2016)") +
  ggtitle("Comparison between Alfonso-Parra et al (2016)\n and current study")
```
